# Surfing the limits of cyanine photocages one step at a time†

**DOI:** 10.1039/d4sc07165d

**Published:** 2024-11-11

**Authors:** Hana Janeková, Sergey Fisher, Tomáš Šolomek, Peter Štacko

**Affiliations:** a Department of Chemistry, University of Zurich Winterthurerstrasse 190 CH-8057 Zurich Switzerland peter.stacko@uzh.ch; b Van't Hoff Institute for Molecular Sciences, University of Amsterdam Science Park 904 1098 XH Amsterdam The Netherlands t.solomek@uva.nl

## Abstract

Near-infrared light-activated photocages enable controlling molecules with tissue penetrating light. Understanding the structural aspects that govern the photouncaging process is essential to enhancing their efficacy, crucial for practical applications. Here we explore the impact of thermodynamic stabilization on contact ion pairs in cyanine photocages by quaternarization of the carbon reaction centers. This strategy enables the first direct uncaging of carboxylate payloads independent of oxygen, resulting in a remarkable two-orders-of-magnitude enhancement in uncaging efficiency. Our computational analyses reveal that these modifications confer a kinetic instead of thermodynamic effect, reducing ion–ion interactions and allowing complete separation of free ions while inhibiting recombination. We demonstrate that, while thermodynamic stabilization is effective in traditional chromophores operating at shorter wavelengths, it rapidly reaches its thermodynamic limitations in NIR photocages by compromising the photocage stability in the dark. Thanks to these findings, we establish that activation of cyanine photocages is limited to wavelengths of light below 1000 nm. Our work illuminates the path to improving uncaging cross-sections in NIR photocages by prioritizing kinetic trapping and separation of ions.

## Abstract 2

Near-infrared light-activated photocages enable controlling molecules with tissue penetrating light.

## Graphical abstract

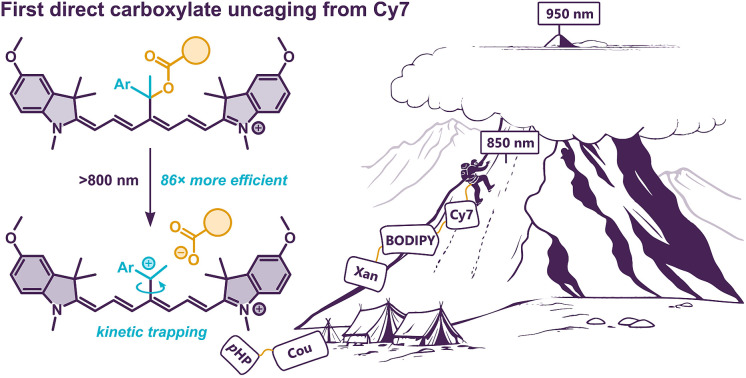

## Introduction

Similar to the cell machinery that regulates biomolecular functions through intricate cascade systems,^1^ photocages offer control over substrate activity precisely in time and space using light as a bioorthogonal stimulus.^2,3^ Photocages can be used to control proteins,^4,5^ nucleotides,^6,7^ drugs,^8,9^ and other biologically significant molecules,^10,11^ which positions them as prime contenders for photoactivated chemotherapy (PACT),^12^ a strategy that complements the photodynamic therapy already exercised in healthcare.^13^ This photochemical tool experienced remarkable development in the past decade driven by the availability of seminal design guidelines^14,15^ that allowed transforming known families of chromophores absorbing biologically benign visible photons, such as BODIPY,^16–24^ porphyrine,^25^ or rhodamine,^26^ into efficient uncaging systems. However, using photocages in PACT requires that the photochemical activation can be achieved non-invasively in deeper organ tissues with a limited access to deliver the light even with optical fibers. Therefore, the light absorption of photocages must reach well into the tissue transparent window that opens in the near-infrared (NIR) region.^27^ Historically, the success of such endeavour remained an open challenge due to a widespread assumption that the subpar excitation energy is insufficient to directly break covalent bonds. However, the introduction of *p*-hydroxyphenacyl (*p*HP)^28–32^ and coumarin^30,33–35^ photocages that operate *via* bond heterolysis mechanisms largely contributed to addressing this conceptual obstacle. Yet, the low energy of the excitated states of one-photon NIR-excitable chromophores comes at an expense of suppressing the efficiency of payload release due to shortened excited state lifetimes.^2,21,22,36^

**Fig. 1:**
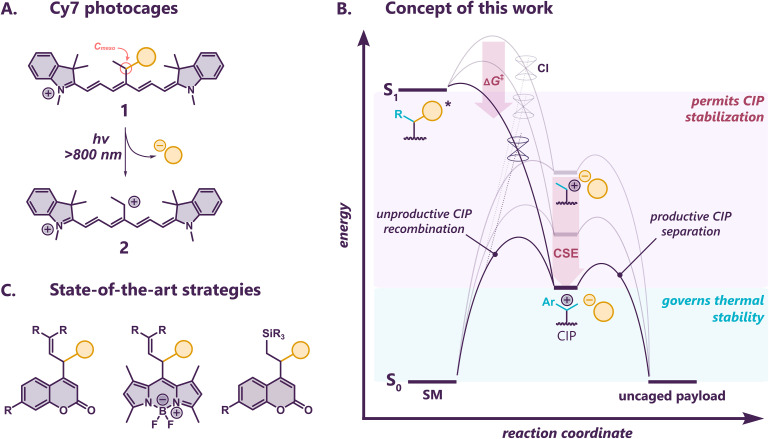
(A) Schematic representation of release from Cy7 photocages, payload is depicted in yellow. (B) Schematic reaction coordinate diagram of the photorelease from Cy7 photocage. SM = starting material, CI = conical intersection, CIP = contact ion pair, CSE = cation stabilization energy (see the ESI† for details). (C) Concepts to increase uncaging performance in the published photocages.

Nevertheless, some of us^37^ and others^38–40^ have recently demonstrated that Cy7 fluorescent dyes can operate as successful photocages (Fig. 1A) with *λ*_max_ exceeding 800 nm, although the observed quantum yields are generally low. Yet, a diverse array of payloads, including carboxylates, amines, phenols and thiols, masked as esters, carbonates and carbamates can be uncaged in biological settings *via* a competition of two mechanisms – photooxidation and photoheterolysis.^37,41^ Direct uncaging avoids dependence of uncaging efficiency on the levels of oxygen, particularly in hypoxic environments such as solid tumors. Unfortunately, a direct photoheterolysis of carboxylates (Fig. 1A) is negligible, *i.e.*, the presence of oxygen to facilitate the photooxidation is inevitable for the release.^37^ The implementation of these Cy7 photocages in control of live human cardiomyocytes^41^ and concurrent delivery of payloads with a subcellular precision^42^ underscore their broad application potential. The recent success of the few NIR-activatable photocages inspires an enticing prospect to reach the short wave infrared window (SWIR, 1000–1500 nm), which would allow reaching unmatched resolution in space and time, and tissue-penetration depth.^27^ Bioimaging has recently pioneered the SWIR territory and showcased its potential by quantification of heartbeat, brain vasculature mapping and multiplexing in live animals.^43–45^ These significant advances beg the questions – can the current photouncaging approaches cross into the SWIR and what are the ultimate limits to reach such an accomplishment?^46^

In this work, we explore the possibility to improve the Cy7 uncaging efficiency by manipulating the stability of the primary carbenium ion formed by heterolysis. Thereby, we achieved for the first time a clean direct uncaging of unmasked carboxylate from Cy7 photocage with a remarkable 100-fold enhancement of the uncaging efficiency. We show that the enhancement is a consequence of kinetic trapping of the primary carbenium ion. We further demonstrate that extending the stabilization strategy reaches the limits of practical use of the photoheterolysis in Cy7 photocages due to inherent thermodynamic boundaries. We discuss how this boundaries affect the available options to improve the uncaging efficiency of NIR photocages by molecular design and propose how to overcome these thermodynamic obstacles.

## Results and discussion

**Scheme 1:**
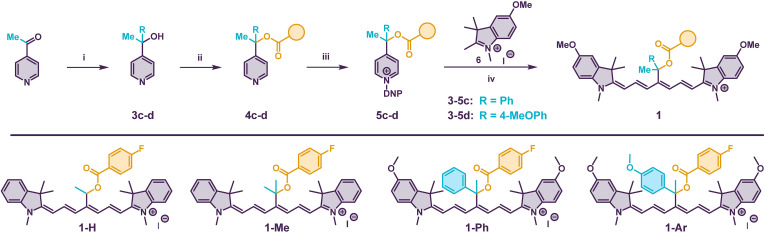
(Top) Synthesis of photocages 1. (i) 3c–d: RMgBr, THF (3c 75%, 3d 61%). (ii) 4c: BuLi, THF, FBA-Cl (69%). 4d: LDA, FBA-Cl THF, −20 °C (64%). (iii) DNP-OTs, acetone (5c 60%, 5d 70%). (iv) AcOK, CH_3_CN or EtOH (1-Ph 30%, 1-Ar 10%). (Bottom) Structure of photocages 1.

Carboxylic acid functional groups are often present in effective medicines. They are involved in specific Coulomb or hydrogen bonding interactions, and are thus often critical for the binding of agents to their targets. Yet their conjugate bases produced at physiological pH increase the hydrophilicity and polarity of the drug, which may impede its bioavailability.^47^ Therefore, many attempts to release carboxylates in controlled manner have been developed.^48,49^ To enable the direct photoheterolysis of carboxylates from Cy7, we decided to revisit the direct uncaging from 1-H (Scheme 1)^37^ that offers additional benefits to photooxidation. It eliminates the variable of varying oxygen levels, which impacts uncaging efficiency, particularly in hypoxic environments such as solid tumors. In addition, photooxygenation approach relies on subsequent diffusion-controlled thermal steps,^40^ limiting its application in studies of fast processes. In our original report, we concluded that direct uncaging from 1-H either does not proceed or it is extremely inefficient. Here, we used a more powerful light source (300 mW cm^−2^) compared to the light source that we used previously (∼40 mW cm^−2^), to irradiate a sample of 1-H under O_2_-free conditions to reveal a sluggish release of 4-fluorobenzoic acid (FBA) in ∼3% chemical yield after 96 h (ESI†). This enabled us to estimate the uncaging quantum yield (*Φ*_rel_) of ∼1.5 × 10^−6^. It should be noted that the value represents the upper bound estimate of the actual value, given the minimal conversion.

The payload release *via* heterolysis from the singlet excited (^1^S*) state of Cy7 and related photocages follows the simplified correlation diagram in Fig. 1B. An excited state barrier separates the ^1^S* minimum from its conical intersection (CI) with the ground state. At the CI, the excited state population branches toward formation of a contact ion pair (CIP) or the starting material (SM). Good nucleofuges in coumarin photocages follow the productive pathway to the CIP with >90% efficiency,^33^ that is, the branching ratio at the CI largely favors heterolysis with only small losses due to unproductive SM regeneration. The dynamics of the bond-breaking process suggests a similar scenario in all related photocage systems. However, a recombination of the primary CIP can have a detrimental effect on the ultimate payload release.^14,34,35^ In the absence of an efficient intersystem crossing, the excited state barrier and/or the ions separation from the CIP then determine *Φ*_rel_.

Compound 1-H operated better *via* direct uncaging in O_2_-free conditions in the presence of a large excess of LiCl (100 mM), although still with only marginal efficacy,^41^ suggesting that interception of the CIP might improve the process. The state-of-the-art strategy to maximize the escape of the ions from the solvent cage is the thermodynamic stabilization of the primary photocage carbenium ion (Fig. 1C).^50,51^ Feringa, Szymański and co-workers demonstrated that a simple vinyl substitution at the *meso*-methylene carbon (C_*meso*_, see Fig. 1A) increases the efficiency of coumarin photocages by 16-fold, and they further showed that this strategy could be successfully extended to BODIPY photocages.^50^ Similar approach using phenyl at C_*meso*_ of BODIPY photocages unexpectedly diminished the release efficiency, possibly due to sterical constrains preventing effective resonance stabilization.^24^ Recently, a strategy using β-silyl effect to increase performance of coumarin photocages has also been investigated.^51^ We first installed an additional methyl substituent to C_*meso*_ in 1-Me to enhance the stability of carbenium ion 2. However, the modest cation stabilization energy (DFT : CSE = 11 kcal mol^−1^, Tables 1, S1 and S2†) was insufficient to expedite the carboxylate release in O_2_-free conditions. We found no conclusive evidence of the release of FBA upon irradiation of 1-Me in MeOD (780 nm) under O_2_-free conditions for 16 hours. In fact, the photolysis showed virtually no conversion within this experimental window like in 1-H.^37^ We thus expected that replacing the extra methyl group for a phenyl ring would exert the necessary stabilization on the carbenium ion.

**Table 1:** Photophysical and photochemical characterization of photocages 1

Photocage	*λ*_abs_ (nm)	*λ*_em_ a (nm)	*ε* (cm^−1^ M^−1^)	*Φ*_F_ a	*Φ*_rel_ c ^,^ h (×10^−5^)	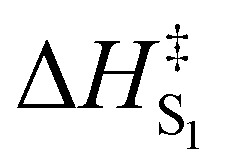 i (kcal mol^−1^)	CSE^g^ (kcal mol^−1^)	Yield%
1-H^d^	786^b^	809	155 400^b^	2.9 ± 0.7	∼0.15	1.3	−4.8	3^e^ (96 h) (51 ± 2^b^)^f^
1-Me^d^	806^a^	843	42 000^a^	<2%	n.d.	0.3	−11.0	n.d. (46 ± 2^b^)^f^
1-Ph	845^a^	878	71 430^a^	<2%	13 ± 2	3.3	−11.1	96 ± 1^e^ (58 ± 3^c^)^f^
1-Ar	846^a^	n.d.	49 830	n.d.	n.d.	3.2	−30.4	n.d.

a Determined in MeOH.

b Determined in PBS (10 mM, pH = 7.4, *I* = 100 mM, 20% DMSO).

c Determined in CD_3_OD.

d Data published in ref. 37.

e Under O_2_-free conditions.

f Under ambient conditions.

g Cation/carbenium ion stabilization energy calculated with DFT using isodesmic reaction approach (Tables S1 and S2).

h Quantum yield of FBA formation determined by ^1^H NMR spectroscopy, (see the ESI for details).

i Heterolysis of pyridinium from Cy7 photocage in the S_1_ state, calculated by TD-DFT (0 K, see the ESI for details). n.d. = not determined.

**Fig. 2:**
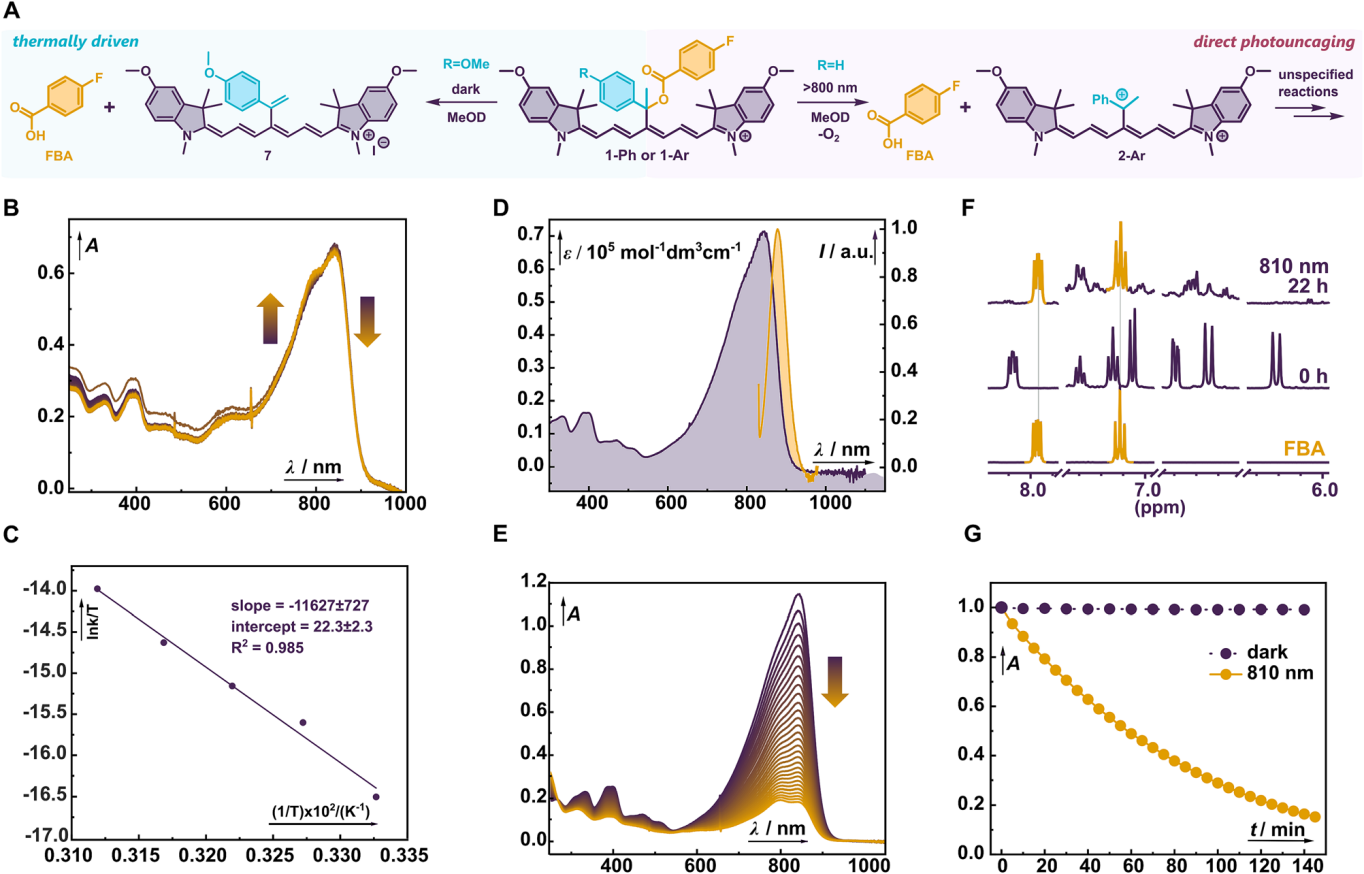
(A) Photouncaging of FBA from 1-Ph under O_2_-free conditions (left) and thermally-driven elimination of FBA from 1-Ar (right). (B) Thermal decomposition of 1-Ar in MeOH followed at rt over 2 hours period by UV-vis spectroscopy (violet to yellow). (C) Eyring analysis of the thermally-driven elimination from 1-Ar. (D) UV-vis (violet) and emission (yellow) spectra of 1-Ph in MeOH. (E) NIR (820 nm) irradiation of 1-Ph in MeOH at ambient conditions, followed by UV-vis spectroscopy (violet to yellow). (F) ^1^H NMR spectra of 1-Ph irradiated in CD_3_OD at 810 nm under O_2_-free conditions. (G) Kinetic traces of 1-Ar irradiated at 820 nm (yellow) and in the dark (violet).

Therefore, we synthesized photocage 1-Ph in five steps using the Zincke chemistry protocol (Scheme 1 and Table 1).^52^ Specifically, we reacted the 4-acetylpyridine with phenyl magnesium bromide providing alcohol 3c in 75% yield.^53^ In the next step, the FBA payload was installed in 69% yield by deprotonation of 3c with BuLi followed by a reaction with the 4-fluorobenzoyl chloride. The reaction of ester 4c with 2,4-dinitrophenyl tosylate in acetone yielded Zincke salt 5c which was subsequently transformed into the final photocage 1-Ph in 30% yield using heterocycle 6 and AcOK in EtOH. Due to its lipophilic nature, 1-Ph strongly aggregates in aqueous media (PBS with up to 50% DMSO as a co-solvent), and MeOH was therefore used in UV-vis spectroscopy studies. The absorption spectrum of 1-Ph with *λ*_max_ = 845 nm is significantly broadened (Fig. 2D) even in MeOH, similar to 1-Me (ref. 37) and other dimethyl analogues reported by Feringa.^38^ We presume that the steric congestion at the reaction center breaks the chromophore symmetry, resulting in crossing the cyanine limit.^54^ Consequently, the absorption coefficients are lower (<80 000 cm^−1^ M^−1^), which negatively impacts the uncaging cross section (*εΦ*_rel_) and applications in multiplexed experiments that call for sharp and narrow spectra. Irradiation of 1-Ph in MeOD (820 nm, 25 mW cm^−2^) for 24 h under ambient conditions led to a complete loss of green color typical for cyanine dyes and a concurrent liberation of FBA observed by ^1^H and ^19^F NMR spectroscopies (ESI†). Complete destruction of cyanine chromophore is in line with our original report of ^1^O_2_-mediated photooxidation of Cy7 photocage followed by a solvolytic release of the payload.^37^ The mediocre chemical yield of the uncaging (∼60%, Table 1) in this case agrees well with previously observed values for structurally related photocages irradiated in the presence of O_2_. To our delight, exclusion of O_2_ revealed that the presence of Ph in 1-Ph does efficiently promote direct photoheterolysis, releasing the payload in excellent 98% chemical yield upon NIR irradiation (810 nm, Fig. 2F). The photolysis of 1-Ph yields a complex mixture of species instead of a defined cyanine photoproduct.

The *Φ*_rel_ of FBA from 1-Ph in O_2_-free conditions was determined to be (1.3 ± 0.2) × 10^−4^. To quantify the influence of the substitution, we calculated the *Φ*_rel_ enhancement *χ* as *Φ*_rel_(1-Ph)/*Φ*_rel_(1-H), which confirms the substantial improvement of uncaging with *χ* = (86 ± 13). Surprisingly, the estimate of the cation stabilization energy (CSE, Table 1) suggests that the replacement of methyl in 1-Me for phenyl in 1-Ph did not markedly affect the energy of the corresponding carbenium ion 2-Ph. The ions 2-Me and 2-Ph are both more stable than that derived from 1-H by ∼6 kcal mol^−1^. Therefore, we investigated (TD-DFT) whether the substitution affected the shape of the ^1^S* potential energy surface decreasing the barrier for heterolysis rather than stabilizing the CIP. Formation of the CIP by photoheterolysis of Cy7 photocages leads to separation of charges between the forming carbenium ion and the conjugate base of the payload. Previous computational works describing the shape of the ^1^S* potential energy surfaces in Cy7 and related photocages using TD-DFT suffer from insufficient mitigation of the associated Coulomb penalty by inaccurate treatment of solvation. Therefore, they provide unrealistic reaction barriers that are insurmountable within the excited state lifetime of the photocages, making comparisons unreliable.^23,38,50^ Description of such a process requires involvement of explicit solvent molecules, which, unfortunately, renders the calculations computationally intractable. For this reason, we employed here a computational protocol that helped us develop the first BODIPY photocage that permitted uncaging large biomolecules in high vacuum.^55^ Briefly, the charge neutral payload is replaced by a pyridinium ion with a p*K*_a_ value close to that of benzoic acid. Photoheterolysis of such pyridinium photocage then shifts the positive charge from the payload to the ensuing primary carbenium ion 2, avoiding charge separation (see Scheme S1†). As a result, this method does not severely suffer from neglecting the solvation and allows to compare a series of potential energy profiles for the same chromophore with different substituents on C_*meso*_.

**Fig. 3:**
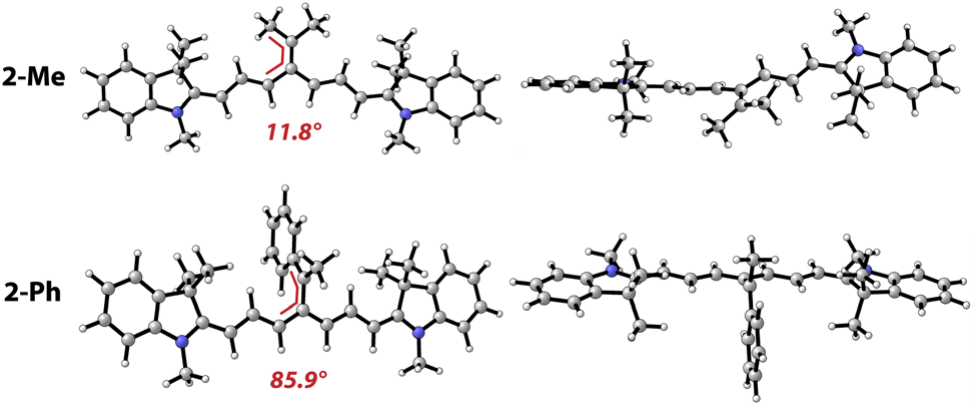
Geometries of carbenium ions 2-Me and 2-Ph: topview (left) and view along C_*meso*_–Cy7 chromophore (right). The angles (in red) denote the rotation of the C_*meso*_ substituents with respect to the Cy7 π-system.

The relaxed potential energy scans for 1-H, 1-Me and 1-Ph obtained by stretching the C_*meso*_–N_*py*_ bond (Fig. S17–S20†) revealed the corresponding reaction barrier after its extension by ∼0.4 Å. The energy then decreases, and the energy surfaces correctly lead to the S_1_/S_0_ CI eventually forming the CIP. Due to absence of the Coulomb penalty, the energies of the computed transition states (Table 1) are markedly lower than in the previous reports.^23,38,50^ For example, an estimate of *Φ*_rel_ for 1-H based on the computed barrier and ignoring CIP recombination predicts *Φ*_rel_ values of ∼10^−1^ suggesting that the method rather underestimates the barriers by a few kcal mol^−1^ (see the ESI† for details). Nevertheless, replacement of methyl in 1-Me for phenyl in 1-Ph did not lower but increased the energy of the computed transition state. We thus tested the effect by pushing the stabilization further with a strong electron-donating methoxy substituent (*σ*^+^ = −0.78, CSE = 30 kcal mol^−1^) in the phenyl *para*-position as in 1-Ar. We found out that the computed barriers for 1-Ph and 1-Ar were similar. All the optimized transition states (Fig. S16†) are relatively early with only a fraction of the positive charge developing on C_*meso*_ (Table S4†). Therefore, the employed substituents cannot develop their thermodynamic stabilization to the full extent experienced in the carbenium ions. In addition, the steric bulk of the aryl substituents appears to contribute to the larger barriers. As a result, the enhancement χ observed for 1-Ph must arise from an effect on the CIP. Inspection of the optimized geometries of the carbenium ions (Fig. 3 and S15†) reveals that those with the aryl substituents differ substantially from 2-Me.

The methyl and aryl groups are perpendicular to the Cy7 chromophore in 2-Ph and 2-Ar due to the steric clash with the four methyl groups in the Cy7 heterocycles. Although they may resemble classical closed-shell 1-arylethan-1-yl carbenium ions, they are diradicaloid in nature^56^ and are markedly less stable (Table S5†). Because the calculated CSEs of 2-Me and 2-Ph are nearly identical, the effect that enhances the *Φ*_rel_ must rather be kinetic than thermodynamic, *i.e.*, the observed rotation likely disrupts the CIP and spatially separates the ions. It is evident by comparing the relative stabilities of 2-Ph and 2-Ar (ΔCSE = 19 kcal mol^−1^) that such separation does not fully dissociate the ions. They must stay loosely connected upon photoheterolysis, otherwise this process in 1-Ph would be endothermic (see below). The potential energy surface of the C_*meso*_ rotation with respect to Cy7 π-system is relatively soft (<5 kcal mol^−1^ by 75°, Fig. S21†) and allows to accommodate the ion of the released payload within a loose CIP to gain extra Coulomb energy. We argue that the dissociation of such looser CIP prevents regeneration of SM that is detrimental to the *Φ*_rel_. Following the rotation coordinate further allowed us to discover a product of 2-Ph rearrangement with comparable energy (1.9 kcal mol^−1^, Fig. S21†). Such rearrangement can explain the formation of complex mixture of products observed by uncaging of FBA from 1-Ph in the absence of O_2_.

In line with our observations, we decided to experimentally probe the additional stabilization in the carbenium ion 2-Ar to see whether a combination of the kinetic and thermodynamic effects can further boost the uncaging efficiency. The synthesis of 1-Ar paralleled that of 1-Ph, but isolation of the compound manifested the limits of the strategy already in the synthetic stage (Scheme 1). Even after extensive and strenuous purification, 1-Ar underwent spontaneous and clean elimination to alkene as evidenced by ^1^H NMR and HRMS spectroscopies (ESI†). The reaction was accompanied by a significant blue shift of the absorption maxima (∼40 nm) congruent with the formation of a cross conjugated system (Fig. 2B). The compromised thermal stability of 1-Ar precluded its in-depth photophysico-chemical investigation. Instead, we opted for an Eyring analysis of the thermal process (Fig. 2C), which uncovered the activation barrier of Δ*G*^‡^ = 24.0 kcal mol^−1^ (25 °C, Δ*H*^‡^ = 23.1 kcal mol^−1^, Δ*S*^‡^ = −2.9 e.u.), corresponding to a half-life of ∼12 h. The observed negative Δ*S*^‡^ is consistent with the data on benzylchloride solvolysis which proceeds *via* S_N_1 mechanism.^57^

The relatively low *Φ*_rel_ of NIR photocages and the short thermal half-life of 1-Ar in solution represent a clear practical limit of the strategies that aim at enhancing the uncaging efficiency by thermodynamic stabilization of the carbenium ion in CIP as we correctly predicted previously^46^ and confirmed here. Synthetic availability of these scaffolds bearing a quaternary reaction center is also a concern. Installation of payload in 1-Ph required harsh conditions – a reaction of an acyl chloride with *in situ* generated alkoxide. In addition, we were unable to install other payloads such as carbonates or carbamates due to immediate elimination in the early stage of the synthesis.

### Reaching the limits

In the following, we evaluate whether uncaging in SWIR region for PACT is thermodynamically feasible. The experimental value of activation barrier for thermal solvolysis (Δ*G*^‡^ = 24.0 kcal mol^−1^ at 25 °C) of 1-Ar can be used to determine the minimal thermodynamic stability of a photocage. The reaction from the S_1_ state must be exergonic by >4 kcal mol^−1^ to avoid equilibrating the CIP with the short-lived S_1_ state. Consequently, the minimum energy of the S_1_ state is 28 kcal mol^−1^ due to the thermodynamic boundaries and corresponds to the maximum uncaging wavelength <1020 nm.‡‡In fact, the maximum uncaging wavelength we provide here is overestimated. To avoid rapid thermal release of a payload, the solvolysis at 37 °C should be ∼10-fold slower than the one in 1-Ar (*t*_1/2_ ∼ 12 h at 25 °C) to permit practical synthesis, handling, administration and biodistribution of the photocage. This gives activation barrier Δ*G*^‡^ = 26.5 kcal mol^−1^ (*t*_1/2_ ∼ 6 days) and a minimum S_1_ energy of 30.5 kcal mol^−1^, which corresponds to the maximum uncaging wavelength <940 nm. Therefore, we argue that achieving direct uncaging in SWIR region with photocages that operate *via* the current photoheterolysis principle in the S_1_ state is practically not viable. The narrow thermodynamic window set by 1-Ar (*E*(S_1_) = 33.2 kcal mol^−1^,§§1-Ar is highly solvolytically (and likely photochemically) unstable. It was not possible to obtain an emission spectrum of 1-Ar; we observed a blue-shifted emission that likely comes from 7 (or a mixture of 7 and 1-Ar). Therefore, we calculated energy of S_1_ state for 1-Ph and expect it is the same (or very similar) for both 1-Ph and 1-Ar. Δ*G*^‡^ = 24.0 kcal mol^−1^) does not provide room for manipulating the energy of CIP to enhance uncaging efficiencies by structural modifications as exercised previously by Feringa, Szymanski and Inose in coumarin and BODIPY photocages (Fig. 1C).^50,51^ While the lower boundary that stems from thermal stability remains the same, these photocages operate at ∼390–520 nm. Consequently, the available energy budget is much larger (27–36 kcal mol^−1^), providing ample room for structural modifications before reaching the stability limits. It is evident that thermodynamic stabilization is not a suitable strategy to improve uncaging efficiencies in NIR photocages. Note that diverting the photoheterolysis from the S_1_ to a triplet state might reach but not cover the entire SWIR region because the photocage thermal stability limit remains. We propose that alternative approaches which introduce a kinetic element, such as in 1-Ph, are sought and tested in the future.

## Conclusions

In summary, we investigated the effect of thermodynamic stabilization of the contact ion pair in cyanine photocages through the quaternarization of their carbon reaction center. The strategy leads to the first direct uncaging of carboxylate payloads from cyanines without a reliance on oxygen. Thereby, we improved the uncaging efficiency by two orders of magnitude. Computational analysis revealed that applied photocage modifications provided a kinetic rather than thermodynamic stabilization to the contact ion pair. This results in loosening the ion–ion interaction facilitating its full separation into free ions and hampering their detrimental recombination. Pushing the thermodynamic stabilization with electron-rich aryl substituent revealed the thermodynamic limits of the approach by impairing the solvolytic stability of the photocage in the dark. This allowed us to address a challenging question concerning the maximum light activation wavelength. We found that the photocages based on cyanine dyes already represent the limit of photouncaging and cannot be activated with light >1000 nm. Further improvement of the uncaging cross-sections in cyanine photocages will require alternative design strategies based on the ion separation through a kinetic trapping like the one introduced here. Such an endeavor is currently ongoing in our laboratories and will be reported shortly.

## Data availability

The data supporting this article have been included as part of the ESI.†

## Author contributions

Hana Janeková – conceptualization, data curation, formal analysis, investigation, methodology, writing – original draft, writing – review & editing; Sergey Fisher – conceptualization, data curation, formal analysis, investigation, methodology, writing – review & editing; Tomáš Šolomek – conceptualization, formal analysis, funding acquisition, project administration, resources, supervision, validation, writing – review & editing; Peter Štacko – conceptualization, formal analysis, funding acquisition, project administration, resources, supervision, validation, writing – review & editing.

## Conflicts of interest

There are no conflicts to declare.

## Supplementary Material

SC-OLF-D4SC07165D-s001

SC-OLF-D4SC07165D-s002
