# Supplementary material for: Surfing the limits of cyanine photocages one step at a time
Source: Chem Sci. 2024 Nov 11;16(4):1677–83. doi: 10.1039/d4sc07165d (PMC11575602; doi:10.1039/d4sc07165d)
Supplement: SC-016-D4SC07165D-s001 [file SC-016-D4SC07165D-s001.pdf]

Supplementary Information (SI) for Chemical Science.
This journal is © The Royal Society of Chemistry 2024

Surfing the Limits of Cyanine Photocages One Step at a Time

Hana Janeková[1], Sergey Fisher[2], Tomáš Šolomek[2,*], Peter Štacko[1,*]

[1]Department of Chemistry, University of Zurich, Wintherthurerstrasse 190, CH-8057 Zurich
[2]Van 't Hoff Institute for Molecular Sciences, University of Amsterdam, Science Park 904, NL-1098 XH Amsterdam, The Netherlands.

Table of Contents

Materials and Methods	S2
Synthesis	S2
Photophysical and Photochemical Measurements Methodology	S5
NMR Spectroscopy	S8
UV-Vis Absorption and Emission Spectroscopy	S18
Plots of Photophysical and Photochemical Measurements	S19
Irradiation Setups	S24
Calculations	S25
References	S34

Materials and Methods

Reagents and solvents of the highest purity available were used as purchased, or they were purified/dried using standard methods when necessary. The intermediates **6**[1], **3c**[2] and photocages **1a** and **1b**[3] were synthesized according to the published procedures or purchased from standard suppliers (Merck, TCI, Across Organics, etc.).

Flash column chromatography was performed using silica gel (230−400 mesh). ^{1}H NMR spectra were recorded on 400 or 500 MHz spectrometers; ^{13}C NMR spectra were obtained on 125 MHz instruments in $CDCl_3$, CD_3OD, and d_6-DMSO. ^{19}F NMR were obtained on 376 MHz or 470 MHz instruments. ^{1}H chemical shifts are reported in ppm relative to $CDCl_3$ (δ = 7.26 ppm), CD_3OD (δ = 3.31 ppm) and d_6-DMSO (δ = 2.50 ppm) as an internal reference. ^{13}C chemical shifts are reported in ppm with $CDCl_3$ (δ = 77.67 ppm), CD_3OD (δ = 49.30 ppm) and d_6-DMSO (δ = 39.52 ppm) as internal references. ^{19}F NMR chemical shifts are reported in ppm either without internal standard, or using C_6F_6 (δ = –165.35 ppm) as an internal reference. Deuterated solvents were kept under nitrogen atmosphere.

Absorption spectra and molar absorption coefficients were obtained on a UV-vis spectrometer with matched 1.0-cm quartz cells. Fluorescence and excitation spectra were measured using a fluorescence spectrometer in a 1.0 cm quartz fluorescence cuvette at 20 °C. The sample concentrations were adjusted to keep the absorbance below 0.2 at the corresponding excitation wavelength. Each sample was measured five times, and the spectra were averaged. Emission and excitation spectra were normalized and corrected by the photomultiplier sensitivity function using correction files supplied by the manufacturer.

The exact masses of the synthesized compounds were obtained using a triple quadrupole electrospray ionization mass spectrometer in a positive or negative mode coupled with direct-inlet.

1-(4-Methoxyphenyl)-1-(pyridin-4-yl)ethan-1-ol (3d)

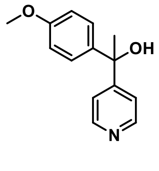

4-Acetylpyridine (12.4 mmol, 1.37 mL) was dissolved in anhydrous THF (30 mL) under inert atmosphere and cooled down to -78°C. A solution of 4-methoxyphenylmagnesium bromide in THF (12.4 mmol, 19.1 mL, 1 M) was added and the mixture was allowed to warm to room temperature over the course of 18 h. Quenched with water (120 mL), extracted with EtOAc (3×150 mL), combined organic phase was dried over $MgSO_4$ and evaporated. The crude product was purified by crystallization (EtOAc/Pentane) to afford the product. Yield 1.73g (61%). White solid. M.p. 140.1–140.5 °C. ^{1}H NMR (500 MHz, d_6-DMSO) δ (ppm) 8.45 (d, J = 5.8 Hz, 2H), 7.38 (d, J = 5.8 Hz, 2H), 7.33 (d, J = 8.7 Hz, 2H), 6.85 (d, J = 8.7 Hz, 2H), 5.84 (s, 1H), 3.71 (s, 3H), 1.80 (s, 3H). ^{13}C NMR (126 MHz, d_6-DMSO) δ (ppm) 158.2, 157.9, 149.2, 140.1, 126.8, 120.6, 113.3, 73.4, 55.0, 29.6. HRMS (ESI+) calcd. for [$C_{14}H_{16}NO_2^+$] 230.1176, found 230.1174.

1-Phenyl-1-(pyridin-4-yl)ethyl 4-fluorobenzoate (4c)

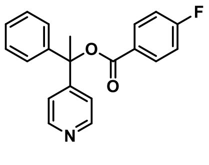

Oxalyl chloride (7.8 mmol, 0.7 mL) and dimethylformamide (1 drop) were added to a suspension of 4-fluorobenzoic acid (580 mg, 3.9 mmol) in dry CH_2Cl_2 (6 mL), at room temperature. The mixture was stirred at room temperature for 30 min. Volatiles were evaporated under reduced pressure and the crude product was used immediately in the subsequent reaction. *n*-BuLi (2.8 mmol, 1.7 mL, 1.6 M in hexanes) was added to a solution of alcohol **3c** (2.5 mmol, 500 mg) in anhydrous THF (20 mL) at 0°C. After stirring for 1 hour, the acyl chloride (prepared

as described above) in THF (2 mL) was added dropwise. The mixture was left stirring at room temperature for 48 h. The reaction was quenched with sat. aq. $NaHCO_3$ (100 mL) and EtOAc (50 mL) was added. The mixture was separated in a separatory funnel, the organic layer was dried with $MgSO_4$ and the volatiles evaporated under reduced pressure. The crude product was purified using column chromatography (SiO_2, pentane: EtOAc gradient 2:1 to 1:1) to afford the product. Yield 558 mg (69%). Orange thick oil. ^{1}H NMR (500 MHz, d_6-DMSO) δ 8.55 (d, J = 6.3 Hz, 1H), 8.24–8.10 (m, 1H), 7.47–7.35 (m, 5H), 7.32–7.24 (m, 1H), 2.29 (s, 1H). ^{13}C NMR (126 MHz, d-$CDCl_3$) δ (ppm) 165.9 (d, J = 254.6 Hz), 163.7, 154.9, 149.5, 143.6, 132.18 (d, J = 9.2 Hz), 128.6, 128.0, 127.1 (d, J = 3.0 Hz), 125.8, 120.9, 115.8 (d, J = 22.0 Hz), 84.2, 26.4. ^{19}F NMR (376 MHz, d-$CDCl_3$) δ (ppm) -105.08. HRMS (ESI+) calcd. for [$C_{20}H_{17}FNO_2^+$] 322.1238, found 322.1240.

1-(4-Methoxyphenyl)-1-(pyridin-4-yl)ethyl 4-fluorobenzoate (4d)

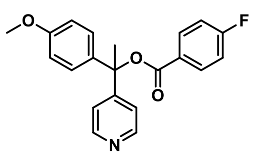

To a suspension of 4-fluorobenzoic acid (7.0 mmol, 1.04 g) in dry CH_2Cl_2 (20 mL), oxalyl chloride (14 mmol, 1.2 mL) and dimethylformamide (1 drop) were added at room temperature. The mixture was stirred at room temperature for 30 min. Volatiles were evaporated under reduced pressure and the crude product was used immediately in the subsequent reaction. n-BuLi (6.11 mmol, 3.8 mL, 1.6 M in hexanes) was added to a solution of the diisopropylamine (6.11 mmol, 0.86 mL) in THF (50 mL) at -20°C. After stirring for 10 minutes keeping the temperature at -20 °C, alcohol **3d** (4.36 mmol, 1.0 g) was added. Following stirring for 15 min, a solution of the acyl chloride prepared above in THF (30 mL) was added dropwise. The mixture was left stirring at room temperature for 18 h. The reaction was quenched with $NaHCO_3$ (sat., aq.) (250 mL) and EtOAc (500 mL) was added. The mixture was separated in a separatory funnel, the organic layer was dried with $MgSO_4$ and the volatiles evaporated under reduced pressure. The crude product was purified using column chromatography (SiO_2, pentane: EtOAc gradient 2:1 to 1:1) to afford the product. Yield 985 mg (64%). Colorless oil. ^{1}H NMR (500 MHz, d-$CDCl_3$) δ (ppm) 8.59 (d, J = 5.5 Hz, 2H), 8.14 – 8.05 (m, 2H), 7.36 – 7.27 (m, 4H), 7.17 – 7.11 (m, 2H), 6.87 (d, J = 8.8 Hz, 2H), 3.80 (s, 3H), 2.29 (s, 3H). ^{13}C NMR (126 MHz, d-$CDCl_3$) δ (ppm) 166.0 (d, J = 254.3 Hz), 163.8, 159.2, 154.8, 150.1, 135.8, 132.2 (d, J = 9.4 Hz), 127.44, 127.40 (d, J = 3.0 Hz), 120.8, 115.8 (d, J = 22.0 Hz), 113.9, 84.3, 55.4, 26.7. ^{19}F NMR (471 MHz, d_6-DMSO) δ (ppm) -105.64. HRMS (ESI+) calcd. for [$C_{21}H_{19}FNO_3^+$] 352.1343, found 352.1343.

General Procedure for the Synthesis of Zincke Salts 5c-d

The corresponding ester **4c-d** (1 eq.) and 2,4-dinitrophenyltosylate (**7**) (1.1 eq.) were suspended in acetone at 40 °C for 18 h. After cooling to room temperature Et_2O (4 mL/mmol) was added, and the product was left to precipitate while stirred for 2 h. The solid was filtered, washed with Et_2O (8 mL/mmol) and dried on air.

1-(2,6-Dinitrophenyl)-4-(1-((4-fluorobenzoyl)oxy)-1-phenylethyl)pyridin-1-ium 4-methylbenzenesulfonate (5c)

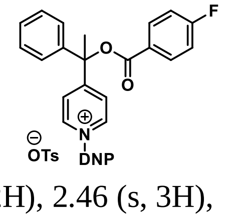

Prepared according to the general procedure from **4c** (400 mg, 1.24 mmol) and 2,4-dinitrophenyltosylate **7** (463 mg, 1.37 mmol). Yield 491 mg (60%). White solid. M.p. 223.1–223.5 °C. ^{1}H NMR (500 MHz, d_6-DMSO) δ (ppm) 9.31 (s, 2H), 9.10 (d, J = 2.5 Hz, 1H), 8.94 (dd, J_1 = 8.7 Hz, J_2 = 2.5 Hz, 1H), 8.56 (d, J = 5.7 Hz, 2H), 8.45 (d, J = 8.7 Hz, 1H), 8.27–8.16 (m, 2H), 7.65 (d, J = 7.5 Hz, 2H), 7.53–7.42 (m, 6H), 7.43–7.37 (m, 1H), 7.10 (d, J = 7.8 Hz, 2H), 2.46 (s, 3H), 2.28 (s, 3H). ^{13}C NMR (126 MHz, d_6-DMSO) δ (ppm) 165.9, 165.6 (d, J = 252.4 Hz), 163.2, 149.2, 146.4, 145.8, 143.2, 141.7, 138.5, 137.6, 132.8 (d, J = 9.5 Hz), 132.0, 130.1, 129.2,

128.6, 128.1, 126.2 (d, J = 2.7 Hz), 125.6, 125.5, 124.2, 121.4, 116.2 (d, J = 22.3 Hz), 83.5, 24.6, 20.8. ^{19}F NMR (471 MHz, d_6-DMSO) δ (ppm) -104.70. HRMS (ESI+) calcd. for [$C_{26}H_{19}FN_3O_6^+$] 488.1258, found 488.1242.

1-(2,6-Dinitrophenyl)-4-(1-((4-fluorobenzoyl)oxy)-1-(4-methoxyphenyl)ethyl)pyridin-1-ium 4-methylbenzenesulfonate (5d)

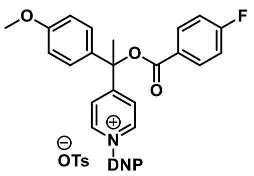

Prepared according to the general procedure from **4d** (500 mg, 1.42 mmol) and 2,4-dinitrophenyltosylate **7** (530 mg, 1.57 mmol). Yield 690 mg (70%). White solid. M.p. 187.5–188.2 °C. ^{1}H NMR (500 MHz, d_6-DMSO) δ (ppm) 9.31 (d, J = 6.7 Hz, 2H), 9.10 (d, J = 2.5 Hz, 1H), 8.94 (dd, J_1 = 8.6 Hz, J_2 = 2.5 Hz, 1H), 8.54 (d, J = 6.5 Hz, 2H), 8.45 (d, J = 8.7 Hz, 1H), 8.20 (dd, J_1 = 8.7 Hz, J_2 = 5.5 Hz, 2H), 7.59–7.53 (m, 2H), 7.50–7.41 (m, 4H), 7.10 (d, J = 7.8 Hz, 2H), 7.03 (d, J = 8.8 Hz, 2H), 3.78 (s, 3H), 2.45 (s, 3H), 2.28 (s, 3H). ^{13}C NMR (126 MHz, d_6-DMSO) δ (ppm) 166.6, 166.0 (d, J = 252.4 Hz), 163.7, 159.6, 149.6, 146.8, 146.3, 143.6, 139.0, 138.0, 134.0, 133.2 (d, J = 9.6 Hz), 132.5, 130.6, 128.5, 127.6, 126.7 (d, J = 2.6 Hz), 126.0, 124.5, 121.8, 116.6 (d, J = 22.1 Hz), 114.8, 83.9, 55.8, 25.0, 21.3. ^{19}F NMR (471 MHz, d_6-DMSO) δ (ppm) -104.81. HRMS (ESI+) calcd. for [$C_{27}H_{21}FN_3O_7$] 518.1364, found 518.1355.

General Procedure for the Synthesis of Cyanines 1c-d

The corresponding Zincke salt **5c-d** (1 eq.) and heterocycle **6** (3 eq) and AcOK (6 eq.) were mixed in MeCN or EtOH (15 mL/mmol). The reaction mixture was stirred at room temperature for 18 h in a flask wrapped in aluminum foil. The volatiles were evaporated under reduced pressure, Et_2O (3 mL/mmol) added, and the precipitate was filtered, dried, washed with Et_2O (6 mL/mmol), H_2O (4 mL/mmol) and Et_2O (2.5 mL/mmol) (if not stated otherwise). The crude product was purified by column chromatography (SiO_2, gradient CH_2Cl_2/MeOH - 100:1 to 30:1, unless stated otherwise).

2-((1*E*,3*Z*,5*E*)-4-(1-((4-Fluorobenzoyl)oxy)-1-phenylethyl)-7-((*E*)-5-methoxy-1,3,3-trimethylindolin-2-ylidene)hepta-1,3,5-trien-1-yl)-5-methoxy-1,3,3-trimethyl-3*H*-indol-1-ium iodide (1-Ph)

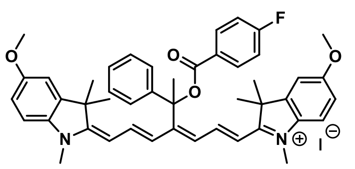

Prepared according to the general procedure in EtOH from **5c** (200 mg, 0.3 mmol) and heterocycle **6** (301 mg, 0.91 mmol). Yield 76 mg (30 %). Dark green solid. M.p. 139 °C (decomp.). ^{1}H NMR (500 MHz, d_4-CD_3OD) δ (ppm) 8.23–8.12 (m, 2H), 7.90 (dd, J_1 = 13.1 Hz, J_2 = 13.5 Hz, 2H), 7.71 (d, J = 7.9 Hz, 2H), 7.52–7.45 (m, 2H), 7.38–7.30 (m, 1H), 7.26–7.19 (m, 2H), 7.15 (d, J = 8.7 Hz, 2H), 7.03 (d, J = 2.4 Hz, 2H), 6.93 (dd, J_1 = 8.7 Hz, J_2 = 2.5 Hz, 2H), 6.80 (d, J = 13.1 Hz, 2H), 6.27 (d, J = 13.5 Hz, 2H), 3.83 (s, 6H), 3.55 (s, 6H), 2.17 (s, 3H), 1.50 (s, 6H), 1.31 (s, 6H). ^{13}C NMR (126 MHz, d_4-CD_3OD) δ (ppm) 172.5, 167.4 (d, J = 253.3 Hz), 165.0, 161.6, 159.8, 147.6, 145.6, 144.1, 138.0, 133.5 (d, J = 9.3 Hz), 130.1, 129.0, 128.4, 126.0, 121.0, 116.7 (d, J = 22.5 Hz), 114.7, 112.3, 109.9, 106.1, 87.2, 56.4, 50.2, 31.7, 29.6, 28.2, 27.9, 17.3. ^{19}F NMR (471 MHz, d_4-CD_3OD) δ (ppm) -107.29. HRMS (ESI+) calcd. for [$C_{46}H_{48}FN_2O_4^+$] 711.3598, found 711.3593.

2-((1*E*,3*Z*,5*E*)-4-(1-((4-Fluorobenzoyl)oxy)-1-(4-methoxyphenyl)ethyl)-7-((*E*)-5-methoxy-1,3,3-trimethylindolin-2-ylidene)hepta-1,3,5-trien-1-yl)-5-methoxy-1,3,3-trimethyl-3*H*-indol-1-ium iodide (1-Ar)

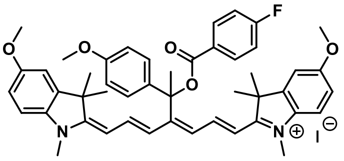

Prepared according to the general procedure in MeCN from **5d** (50 mg, 0.07 mmol) and heterocycle **6** (72 mg, 0.22 mmol). The reaction mixture was diluted with CH_2Cl_2, extracted with brine (100 mL), combined organic layer was dried over $MgSO_4$ and volatiles were evaporated under reduced pressure. Crude product was purified by column chromatography (SiO_2, gradient CH_2Cl_2 (0.3% Et_3N) to CH_2Cl_2(0.3% Et_3N) /MeOH 50:1). Solid after evaporation was suspended in Et_2O (50 mL) and filtered, washed with Et_2O (3×30 mL) and dried. Yield 6 mg (10 %). Dark green solid. ^{1}H NMR (500 MHz, d_3-CD_3CN) δ (ppm) 8.18–8.14 (m, 2H), 7.83 (dd, J_1 = 13.2 Hz, J_2 = 13.4 Hz, 2H), 7.63–7.59 (m, 2H), 7.20 (dd, J_1 = 8.7 Hz, J_2 = 8.6 Hz, 2H), 7.09 (d, J = 8.7 Hz, 2H), 7.04 – 7.00 (m, 4H), 6.91 (dd, J_1 = 8.6 Hz, J_2 = 2.6 Hz, 2H), 6.76 (d, J = 13.1 Hz, 2H), 6.20 (d, J = 13.5 Hz, 2H), 3.81 (s, 6H), 3.79 (s, 3H), 3.46 (s, 6H), 1.46 (s, 6H), 1.30 (s, 6H), 1.28–1.26 (m, 3H). ^{13}C NMR (126 MHz, d_3-CD_3CN) δ (ppm) 166.8 (d, J = 252.2 Hz), 164.3, 160.6, 160.2, 159.0, 144.7, 143.9, 139.5, 137.8, 133.3 (d, J = 9.7 Hz), 127.2, 120.4, 116.6 (d, J = 22.1 Hz), 115.1, 114.4, 112.3, 109.9, 105.9 (d, J = 3.4 Hz), 86.7, 66.3, 56.6, 56.1, 47.3, 32.2, 30.1, 29.7, 27.9, 27.7. ^{19}F NMR (471 MHz, d_3-CD_3CN) δ (ppm) -106.47. HRMS (ESI+) calcd. for [$C_{47}H_{50}FN_2O_5^+$] 741.3704, found 741.3705. The purity of the compound was severely compromised due to extremely low solvolytic stability. Changing the heterocycles to *des*-OMe analogues had no influence on the stability.

Photophysical and Photochemical Measurements Methodology

UV-Vis Absorption and Emission Measurements.

Fluorescence Measurements.
Emission spectra were measured in methanol using a fluorescence spectrometer in a 1.0 cm quartz fluorescence cuvette at 20 °C. The sample concentrations were adjusted to keep the absorbance <0.15 at the corresponding excitation wavelength. Each sample was measured five times, and the spectra were averaged. Emission spectra were normalized and corrected by the photomultiplier sensitivity function using correction files supplied by the manufacturer. The fluorescence quantum yields (Φ_F) were determined using integration sphere, each sample was measured five times using independent solutions keeping A<0.15, and the spectra were averaged. In all cases, the Φ_F were below 2% which is the detection limit of the machine in this spectral range.

Irradiation Experiments and Dark Stability.
A solution of photocage **1-Ph–Ar** (c ~1–1.5 × 10^{-5} M, 3100 μL, A < 1.5) in methanol was stirred and left to equilibrate for 2–3 min at 20 °C under ambient conditions. Afterward, the sample was irradiated with LEDs at 820 nm (~25 mW/cm^2) and the progress of the irradiation was monitored at the given time intervals by UV-vis spectrometry. The total irradiation time was selected to reach >95% conversion and to obtain minimum of 30 experimental points. The procedure was repeated three times. The dark stability of **1c–d** was recorded using the same procedure with exclusion of the irradiation source. For **X** only dark stability in MeOH was measured due to low thermal stability.

Decomposition Quantum Yield Determination.

A solution of photocage **1-H-Ph** ($c < 1 \times 10^{-5}$ M, 3100 µL, A < 1.5) in MeOH was stirred and left to equilibrate for 2–3 min at 20 °C under ambient conditions. Afterward, the sample was irradiated with a beam of collimated light at 780 nm (Ø=7 mm) and UV-vis spectra were recorded periodically using diode-array spectrophotometer. The radiant power (flux; Φ_e) of the light source was determined using calibrated Si-photodiode and optical power meter (~40 mW). The total irradiation time was selected to reach <10% conversion of the photocages and to obtain ten experimental points. The procedure was repeated three times. The quantum yield of decomposition $\boldsymbol{\Phi}_{\mathbf{dec}}$ was calculated according to the equation:

$$\phi_{\mathrm{dec}} = \frac{\Delta n_{dec}}{\Delta n^{p}_{abs}} \qquad \text{(Eq. 1)}$$

where Δn is the number of moles of the photodecomposed photocage **1-H-Ph** calculated from the absorbance change at $\lambda_{\max}$, and Δn^{p}_{abs} is the number of moles of photons absorbed by the sample in the give time period, calculated according to the equation:

$$\Delta n^{p}_{abs} = \frac{\int_0^t \int_0^\infty (1 - 10^{-A(\lambda,t)})\; I_\lambda^{em}\; d\lambda\; dt}{6.022 \times 10^{23}} \qquad \text{(Eq. 2)}$$

where $A(\lambda,\mathrm{t})$ is the absorbance of the sample at the wavelength λ in time t, and I_λ^{em} is the photon flux of the LED source at the wavelength λ determined according to the equation:

$$I_\lambda^{em} = q_n(\lambda)\; \frac{e}{\int_0^\infty \frac{hc}{\lambda}\; q_n(\lambda)\; d\lambda} \qquad \text{(Eq. 3)}$$

where $q_{\mathrm{n}}(\lambda)$ is the emission spectrum of the LED source provided by manufacturer (counts vs. wavelength) and Φ_e is the radiant power (flux) measured by the optical power meter.

Irradiation Followed By NMR spectroscopy and Chemical Yield Quantification Experiments.

Photocage **1-Ph** (~0.6–0.8 mg) was dissolved in aerated or degassed d_4-CD_3OD (0.5 mL). Methanol for experiments under oxygen-free conditions was extensively degassed by 5 freeze-pump-thaw cycles using liquid N_2 and the samples were prepared in a glovebox. For the purpose of chemical yield quantification, C_6F_6 and TMSB (1,4-bis(trimethylsilyl)benzene) were used as internal standards for ^{19}F NMR and ^{1}H NMR spectroscopy respectively. The NMR tube was then irradiated with a set of LEDs at 810 nm 810 nm (~300 mW cm^{-2}, at a fixed distance of ~3 cm, cooled by a fan at 1200 rpm) and ^{1}H and/or ^{19}F NMR spectra with extended acquisition times were recorded after indicated time intervals. Total irradiation surface can be approximated as area of a cylinder of the NMR tube filled by solvent (d = 5 mm, l = 40 mm, A ~6.3 cm^{2}), though this value does not pain a full picture due to non-uniformity of light intensity and reflections on the curved surface of the tube. NMR samples after the irradiation under oxygen-free conditions was subsequently analyzed by HRMS upon dilution. In the control dark experiments, samples were prepared as described above and kept in dark throughout the duration of the experiment.

Quantum Yield of Uncaging via Photoheterolytic Pathway.

Photocage **1-H, 1-Ph** (1.83–1.85 mg) was dissolved in degassed d_4-CD_3OD (0.6 mL) and quantitatively transferred into NMR tube in glovebox. d_4-CD_3OD for this experiment was

prepared as described above and contained same internal standards. The NMR tube was then irradiated with an LED light source at 810 nm (~300 mW cm^{-2}, at a fixed distance of ~3 cm, cooled by a fan at 1200 rpm) and ^{1}H NMR spectra were recorded using extended acquisition times after irradiation for 40 and 60 minutes. As a reference, ICG (indocyanine green) (1.79–1.81 mg) was dissolved in d_4-CD_3OD (0.55 mL) under ambient conditions and irradiated using the same LED light source at 810 nm. The quantum yield of decomposition of ICG Φ_{ICG} was estimated using equation:

$$\Phi_{ICG} = \Phi_{\Delta} \frac{k_r[ICG]}{k_d + k_r[ICG]}$$

where the Φ_{Δ} = 0.008, k_r = (8.7±0.5)×10^6 M^{-1} s^{-1} and k_d = 4.41×10^3 s^{-1} in d_4-CD_3OD. [ICG] corresponds to initial concentration of ICG (~4 mM).[4,5]

Graph of Δn_{dec} or $\Delta n_{release}$ vs. time (t (s)) was then constructed and the $\Phi_{release}$ was calculated using equation:

$$\Phi_{release} = \Phi_{ICG} \frac{slope(1a)}{slope(\mathrm{ICG})}$$

where Φ_{ICG} is quantum yield of decomposition of ICG calculated above and slope(X) and slope(ICG) are slopes of the linear regression of dependence of Δn_{dec} or $\Delta n_{release}$ vs. time (t (s)).

Both measurements (sample and the reference) were repeated three times and the value $\Phi_{release}$ is given as an average with a standard deviation of the mean.

Activation Barrier for Thermal Decomposition (Eyring Analysis).
Photocage **1-Ar** (0.76–0.95 mg) was dissolved in MeOD (0.6 mL, containing TMSB as a standard for quantification) in dark and ^{1}H NMR spectra were measured immediately at temperatures: 300.6 K, 305.6 K, 310.6 K, 315.6 K, 320.6 K in kinetic mode over the time course necessary to achieve ~30% conversion. Plotting ln c against time for each temperature and determining the slope of the fit provided the respective k (rate constant). The Eyring plot was constructed by correlation ln(k/T) against $1/T$. The activation parameters were calculated using Eyring equation:

$$ln\frac{k}{T} = \frac{-\Delta H^{\#}}{R}.\frac{1}{T}+ln\frac{k_B}{h}+\frac{\Delta S^{\#}}{R}$$

where k is rate constant obtained from each temperature point as described above, T is temperature in K, $\Delta H^{\#}$ is activation enthalpy, R is gas constant, k_B is Boltzmann's constant, h is Planck constant and $\Delta S^{\#}$ is activation entropy.

NMR Spectroscopy

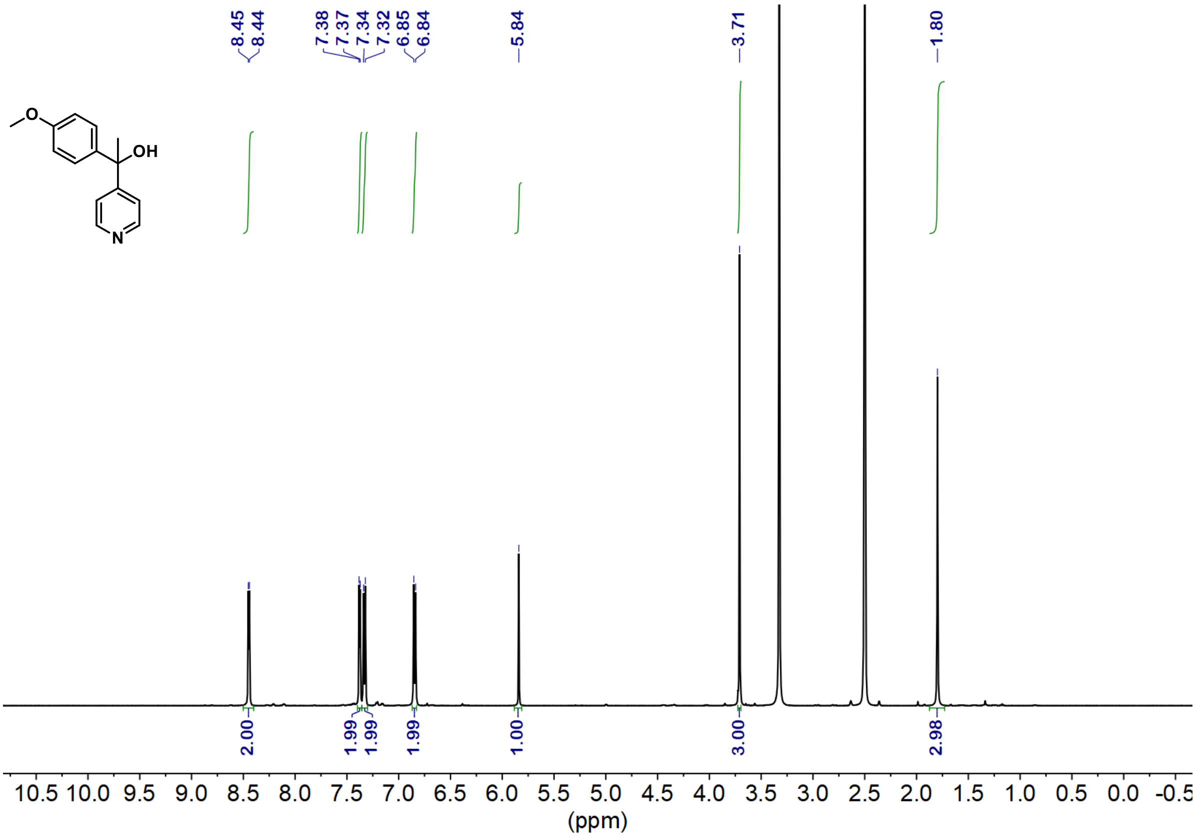

^{1}H NMR (500 MHz, d_6-DMSO) of **3d**

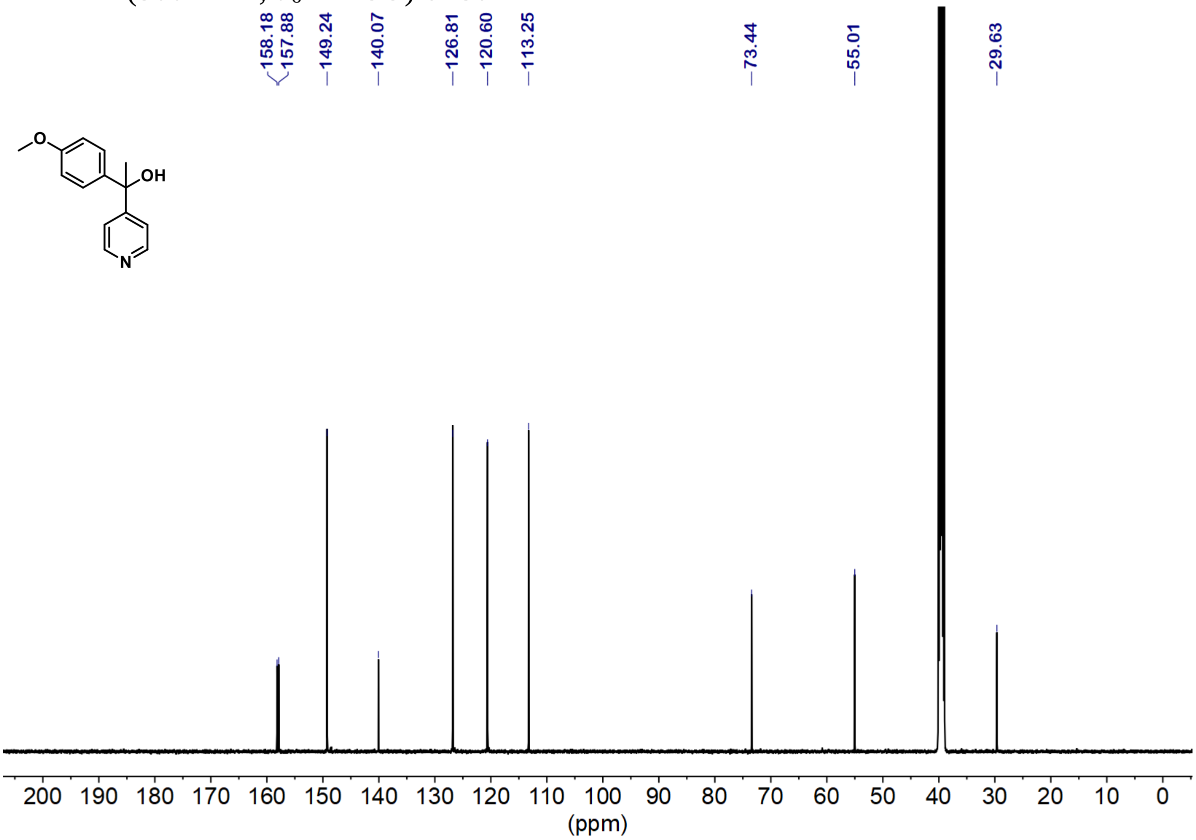

^{13}C NMR (126 MHz, d_6-DMSO) of **3d**

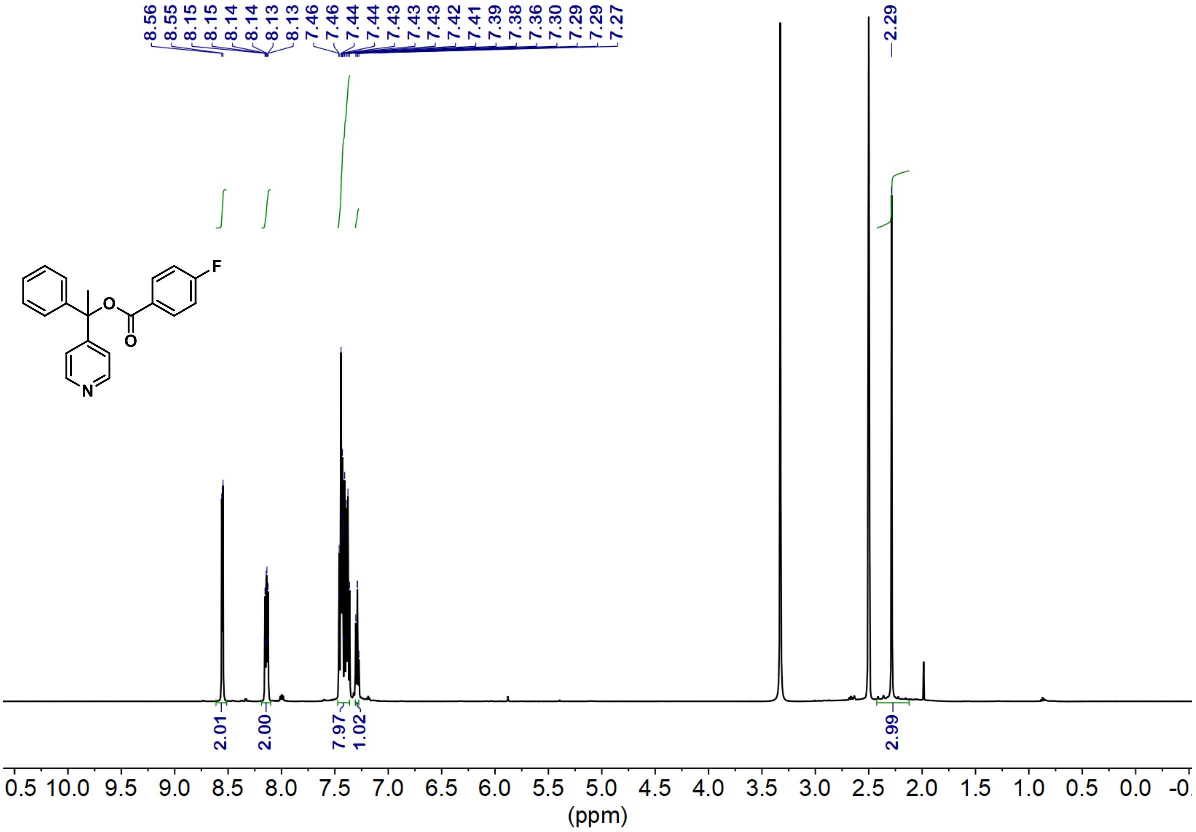

^{1}H NMR (500 MHz, d_6-DMSO) of **4c**

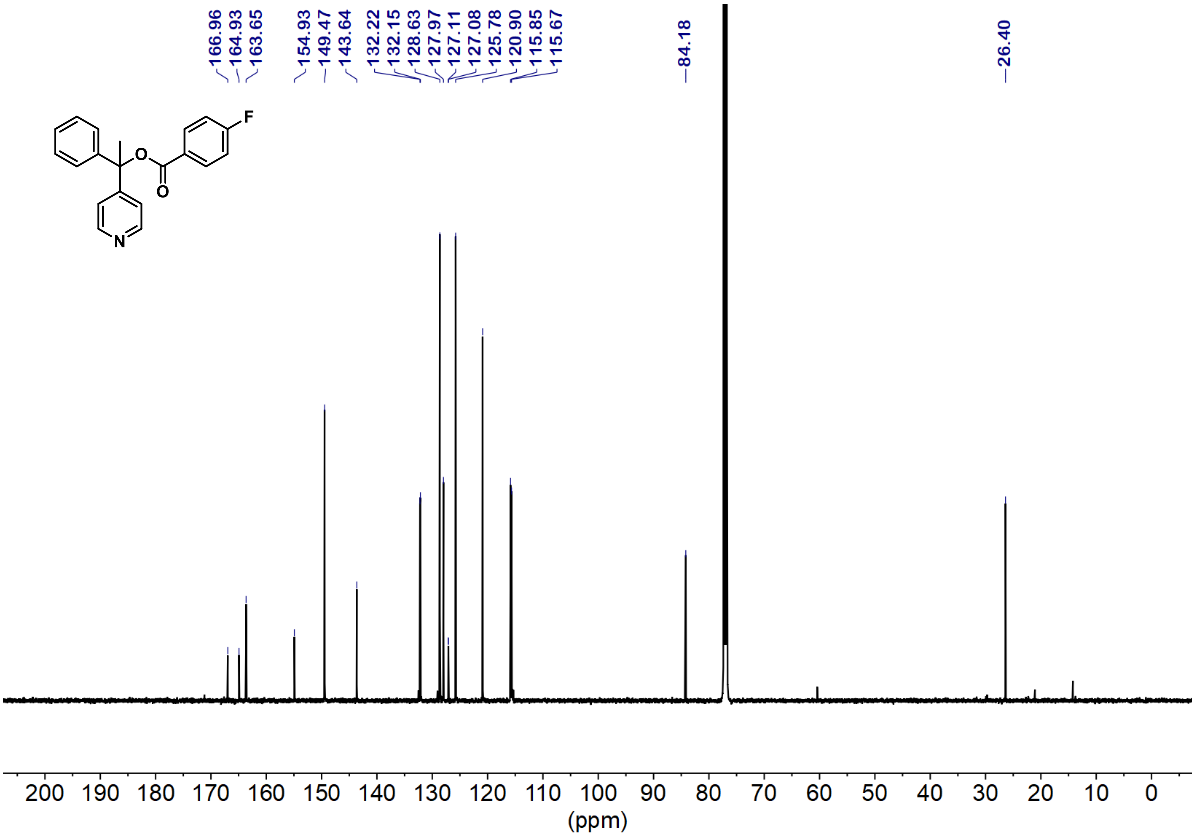

^{13}C NMR (126 MHz, *d*-$CDCl_3$) of **4c**

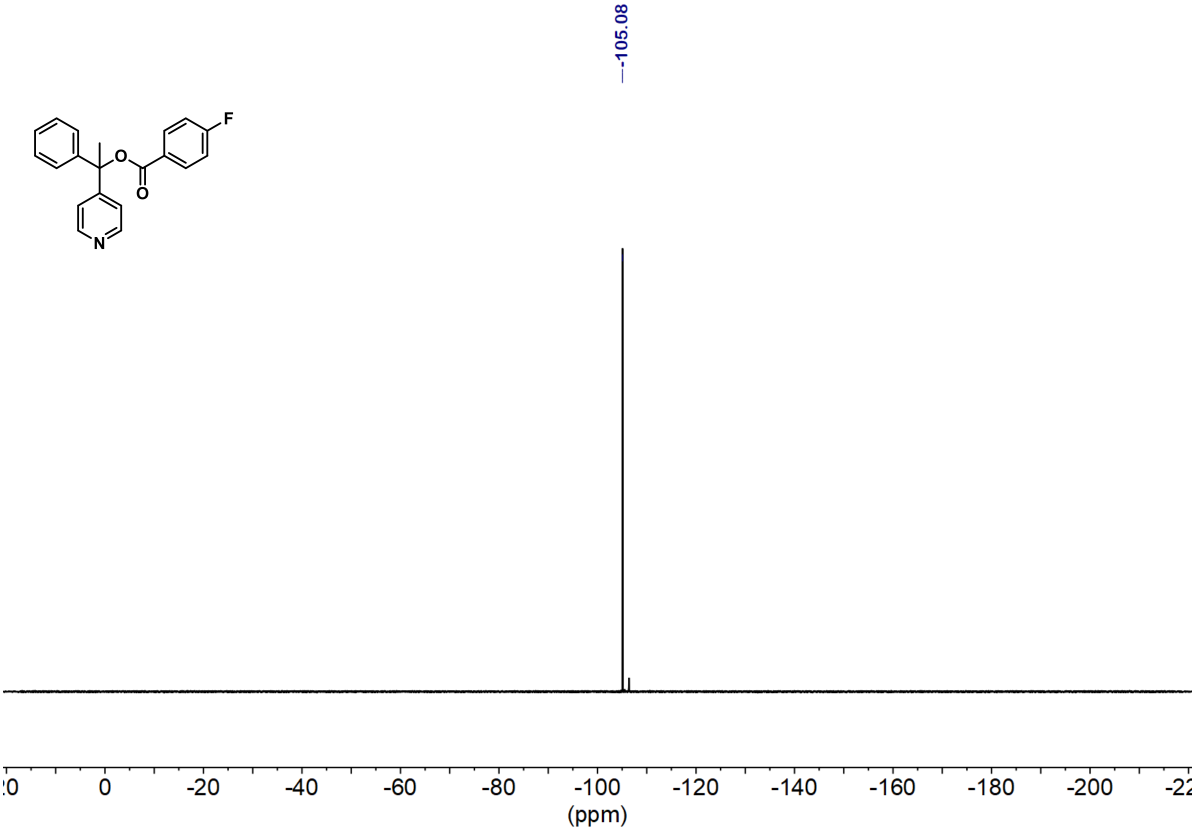

^{19}F NMR (376 MHz, *d*-$CDCl_3$) of **4c**

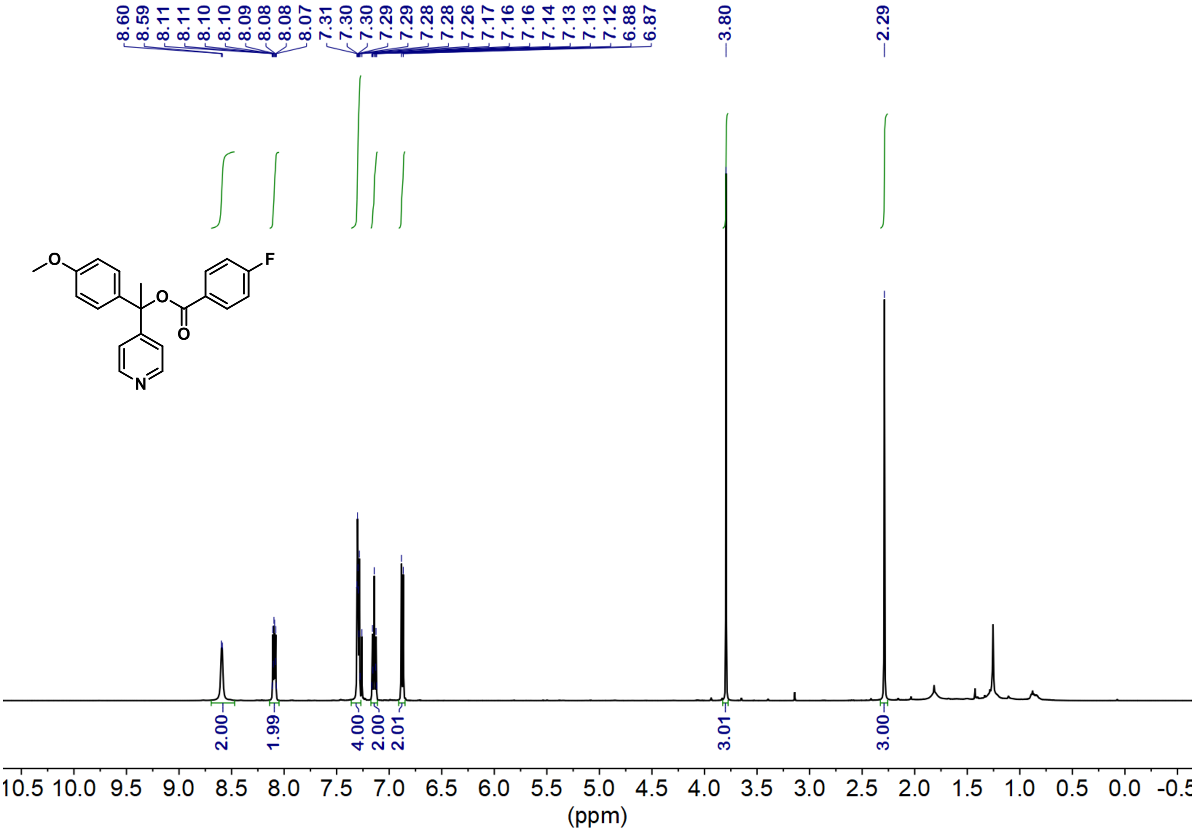

^{1}H NMR (500 MHz, *d*-$CDCl_3$) of **4d**

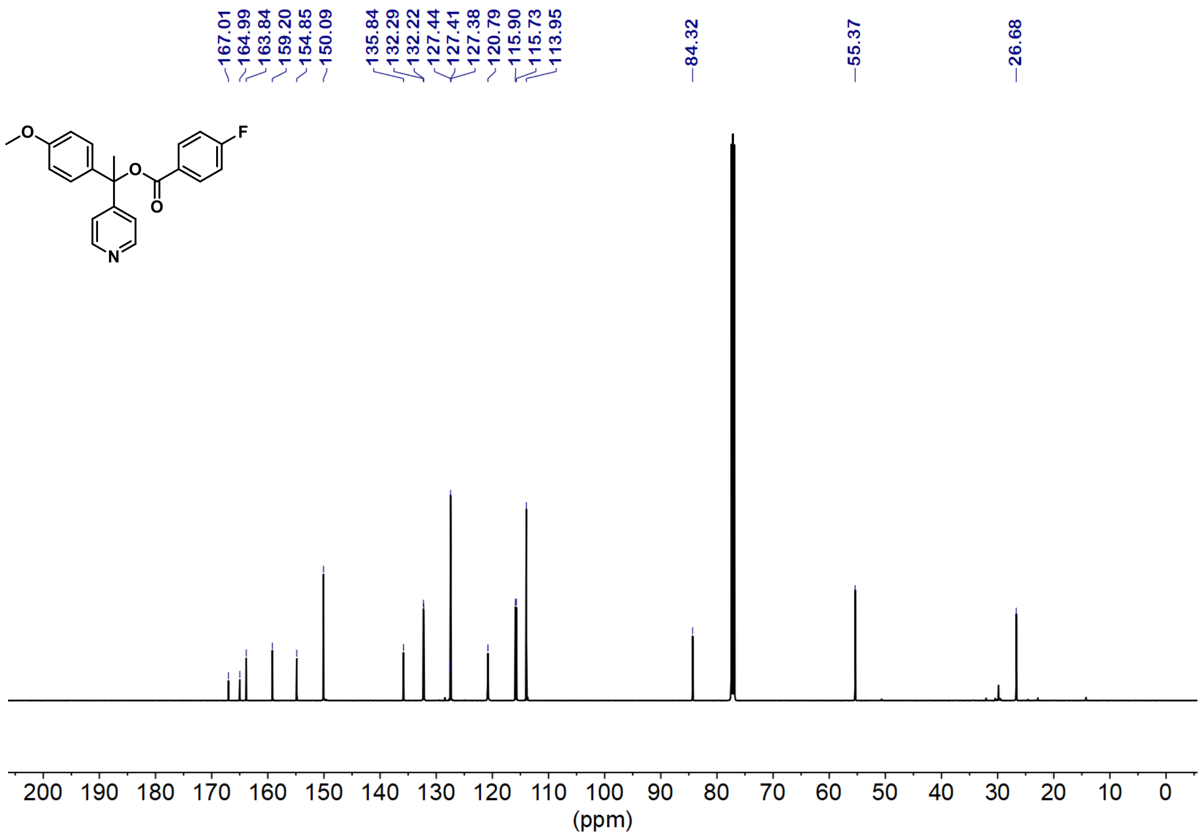

^{13}C NMR (126 MHz, *d*-$CDCl_3$) of **4d**

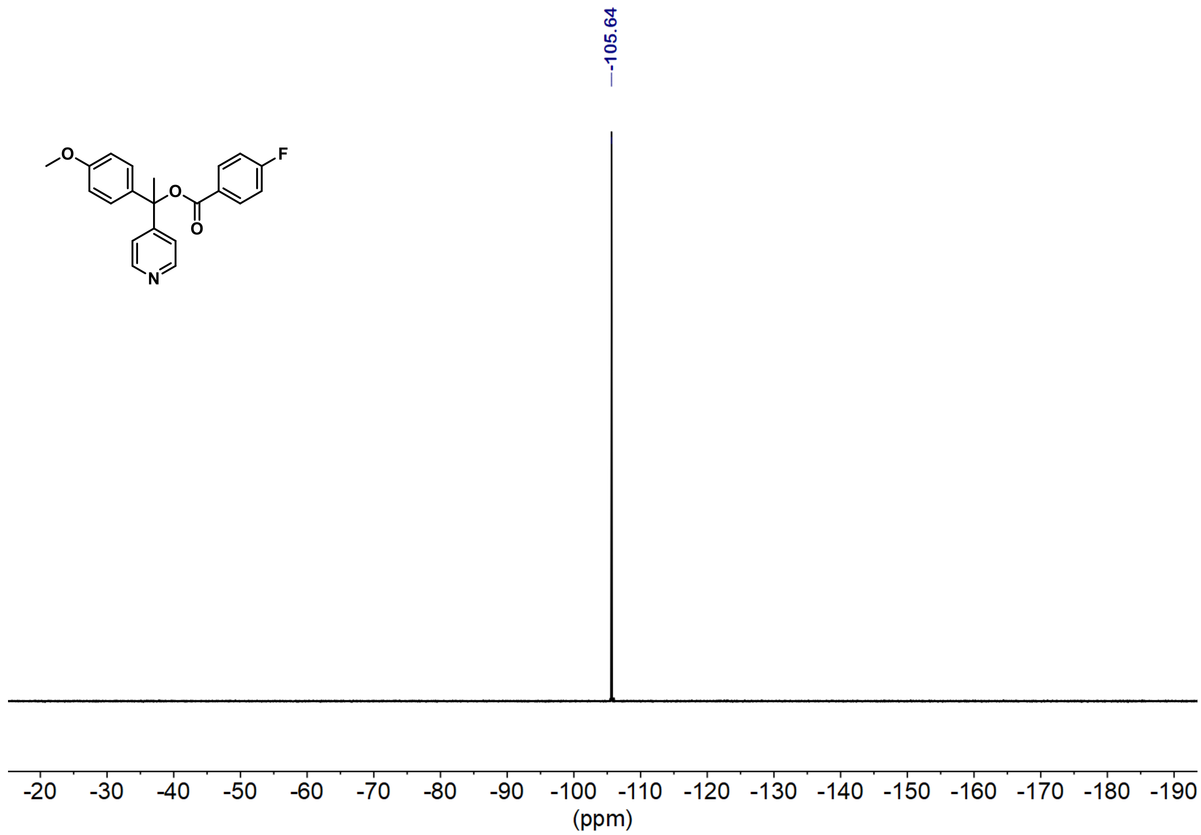

^{19}F NMR (471 MHz, d_6-DMSO) of **4d**

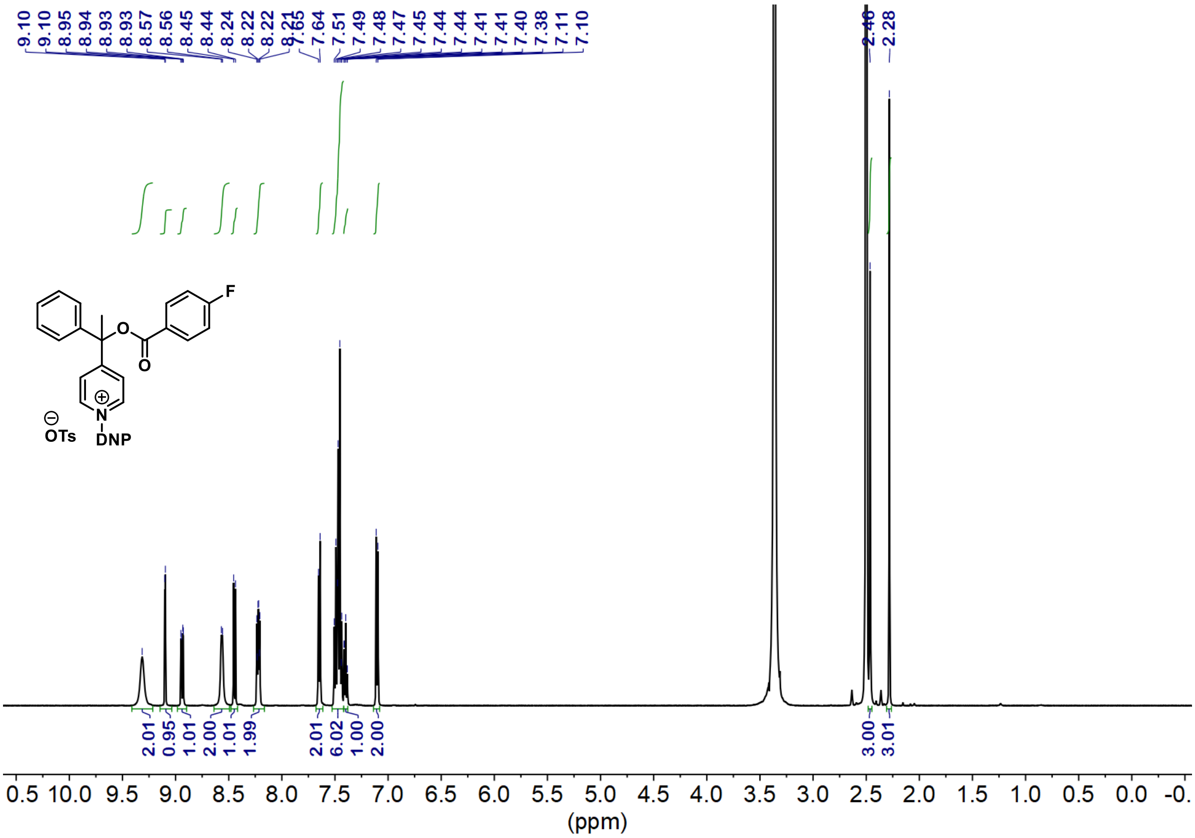

^{1}H NMR (500 MHz, d_6-DMSO) of **5c**

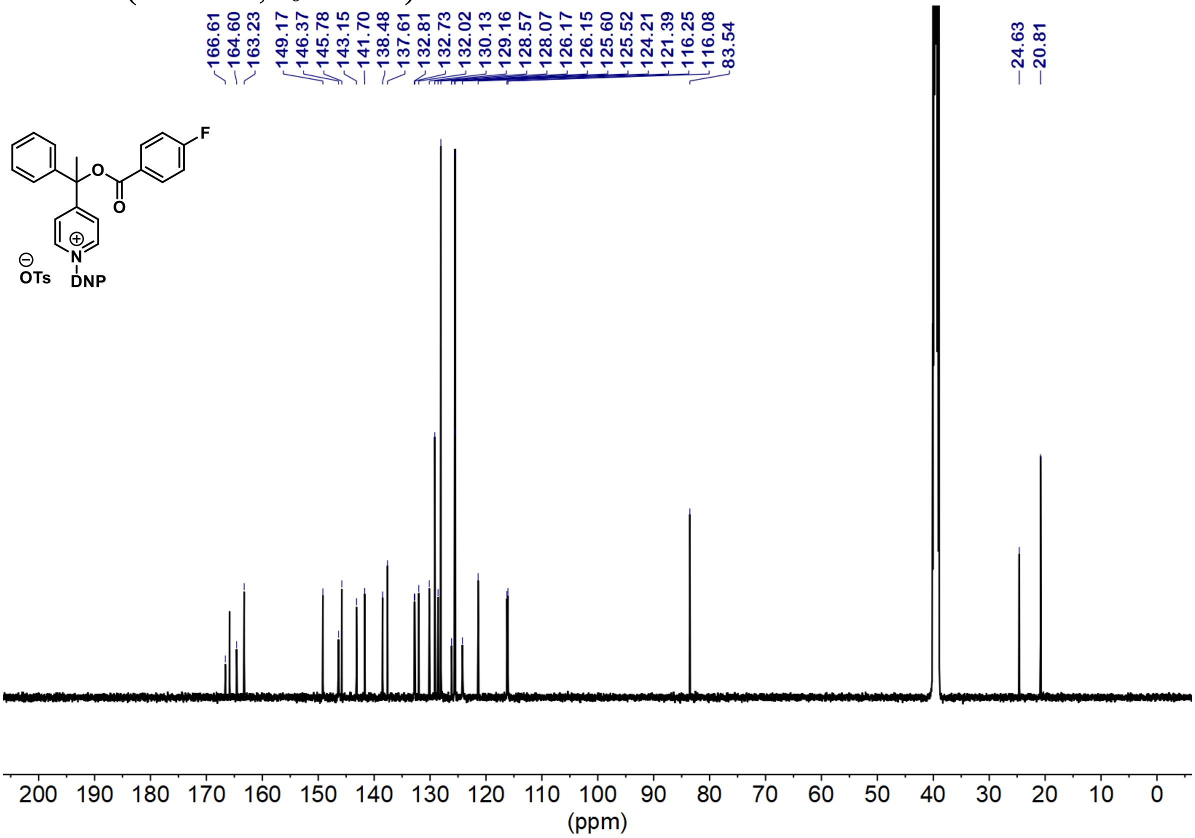

^{13}C NMR (126 MHz, d_6-DMSO) of **5c**

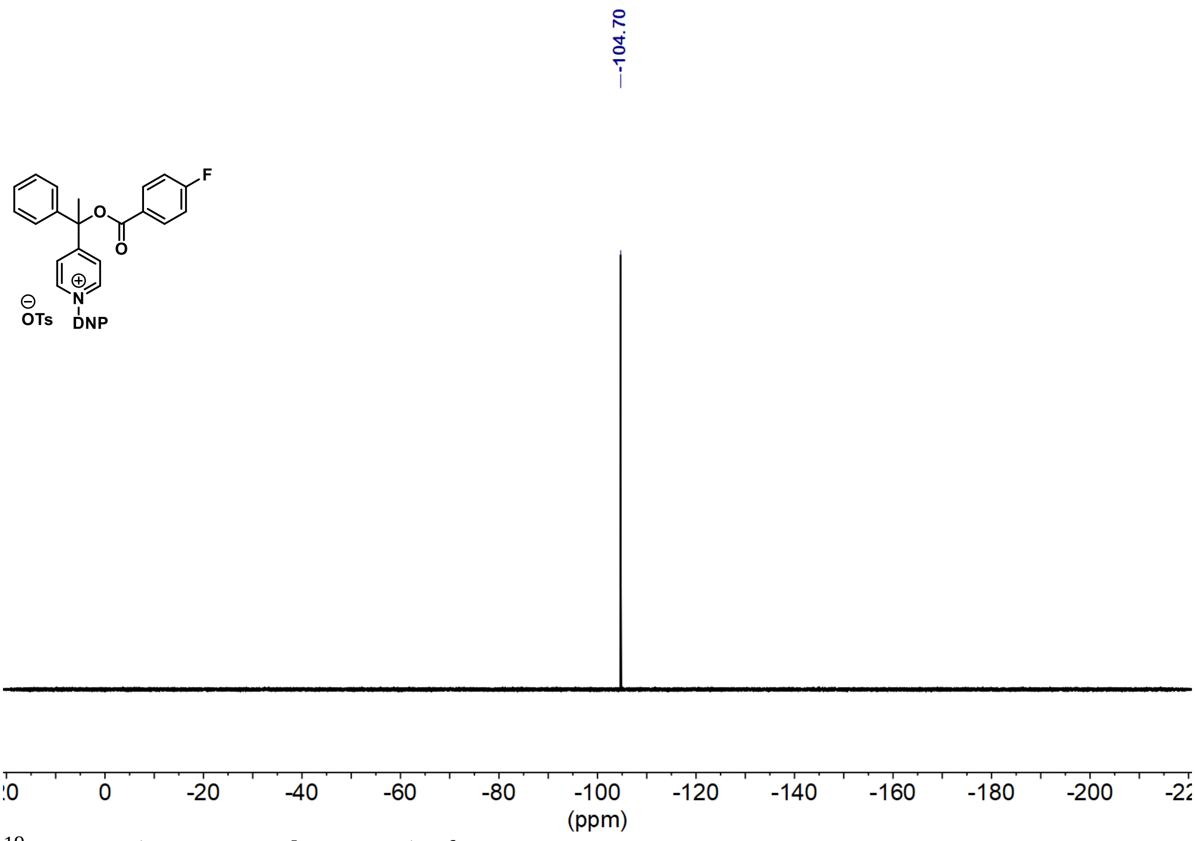

^{19}F NMR (471 MHz, d_6-DMSO) of **5c**

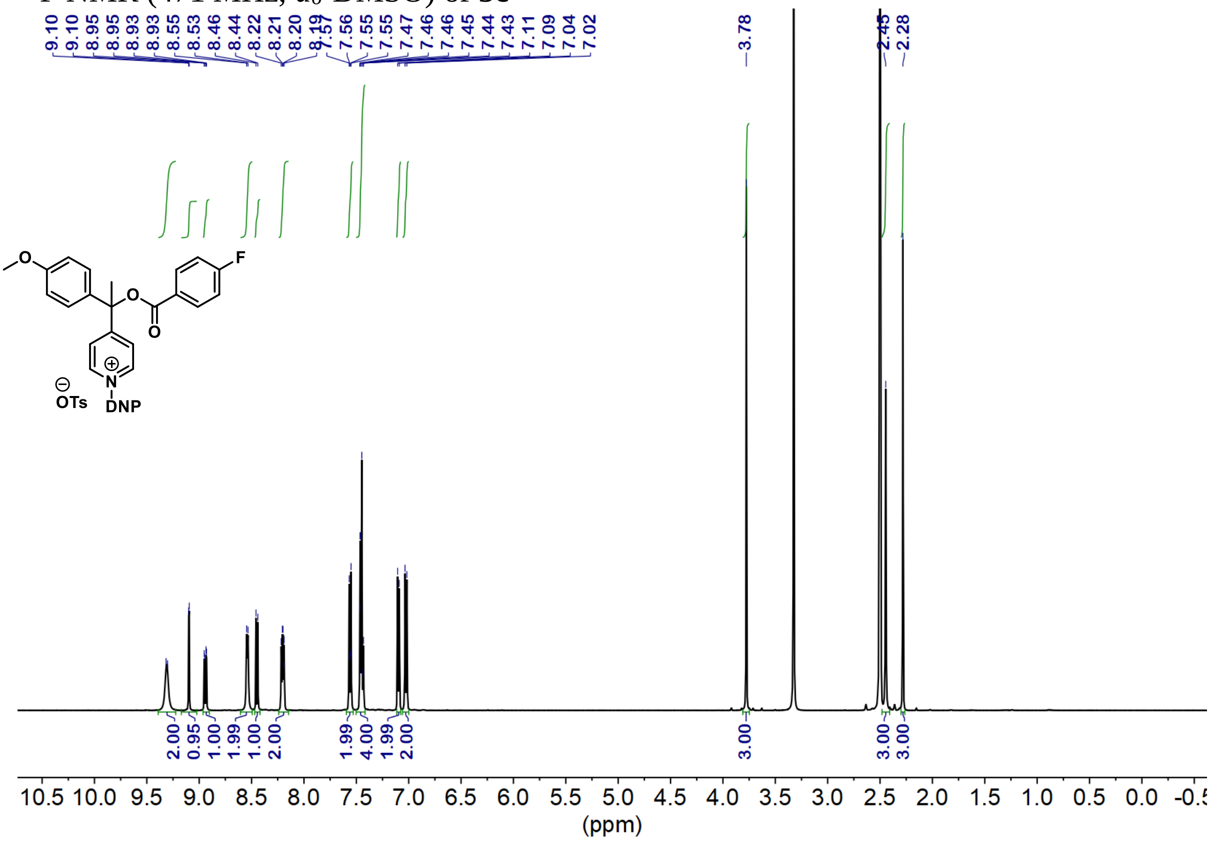

^{1}H NMR (500 MHz, d_6-DMSO) of **5d**

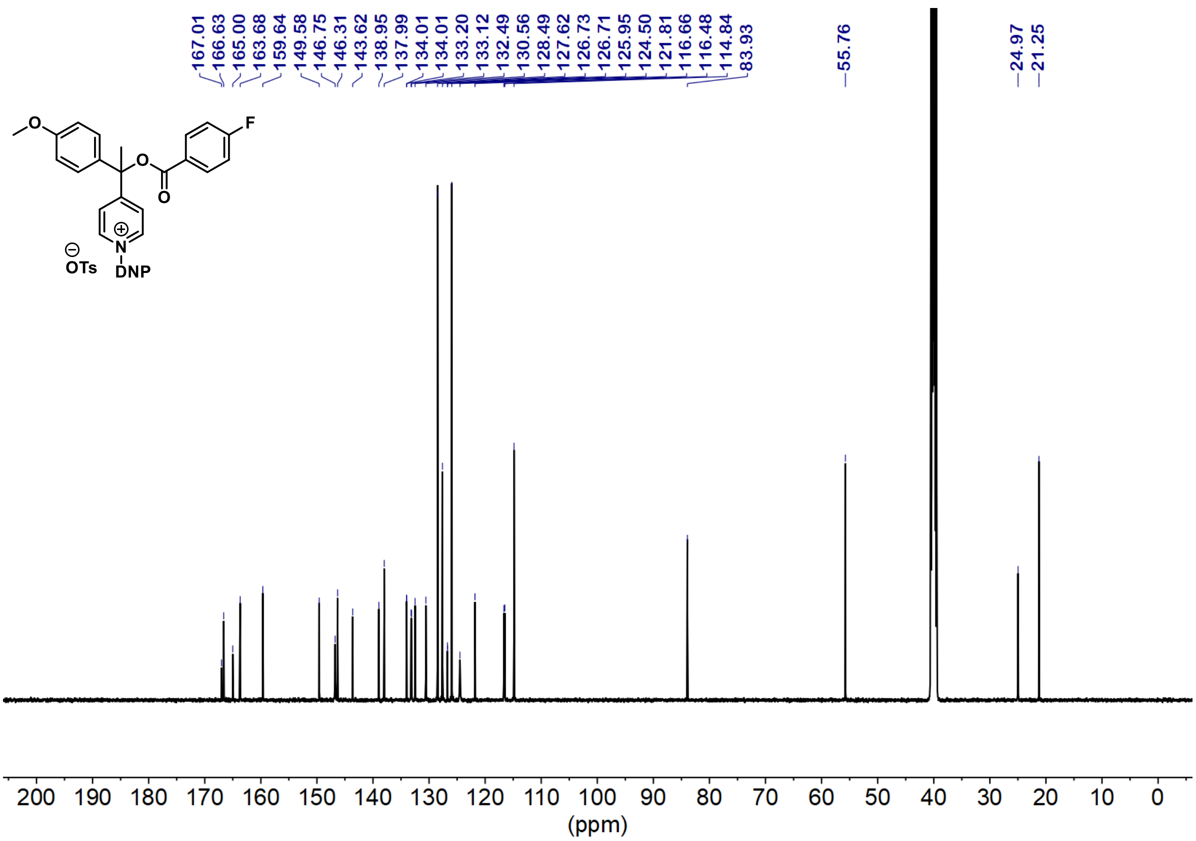

^{13}C NMR (126 MHz, d_6-DMSO) of **5d**

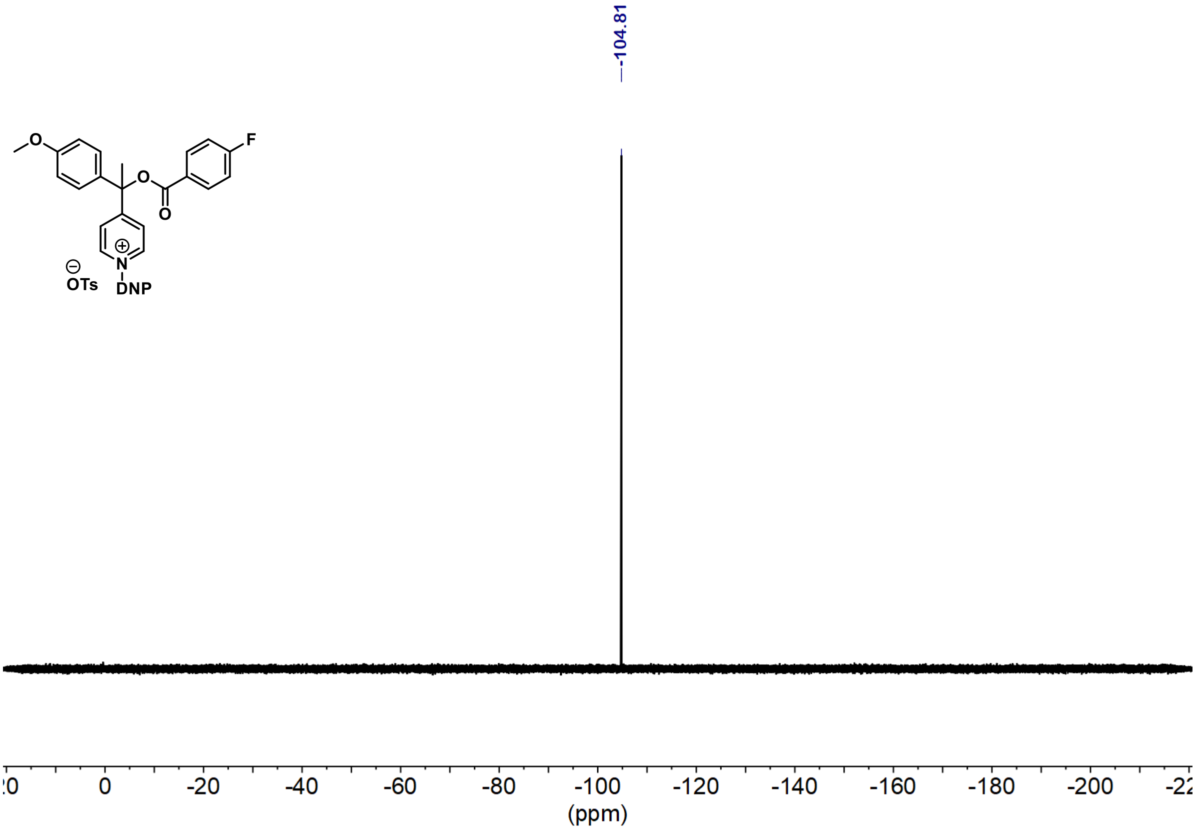

^{19}F NMR (471 MHz, d_6-DMSO) of **5d**

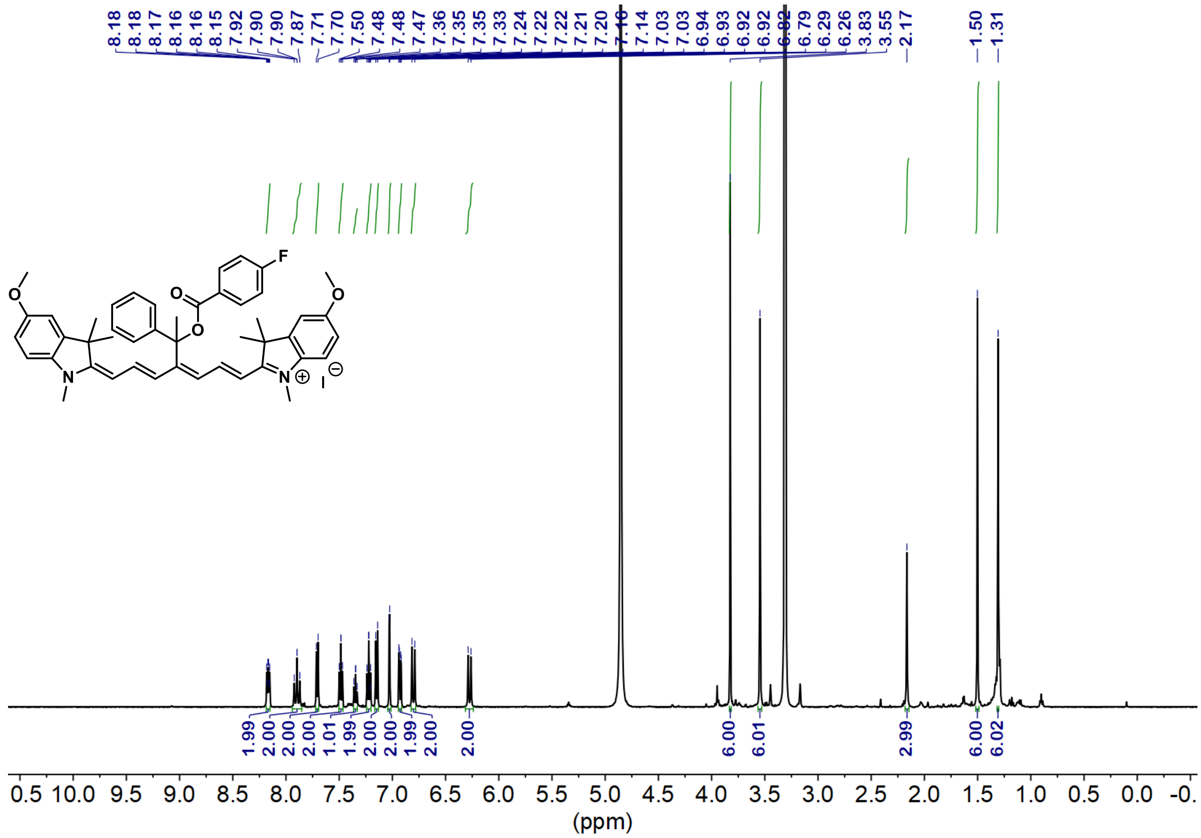

^{1}H NMR (500 MHz, d_4-CD_3OD) of **1-Ph**

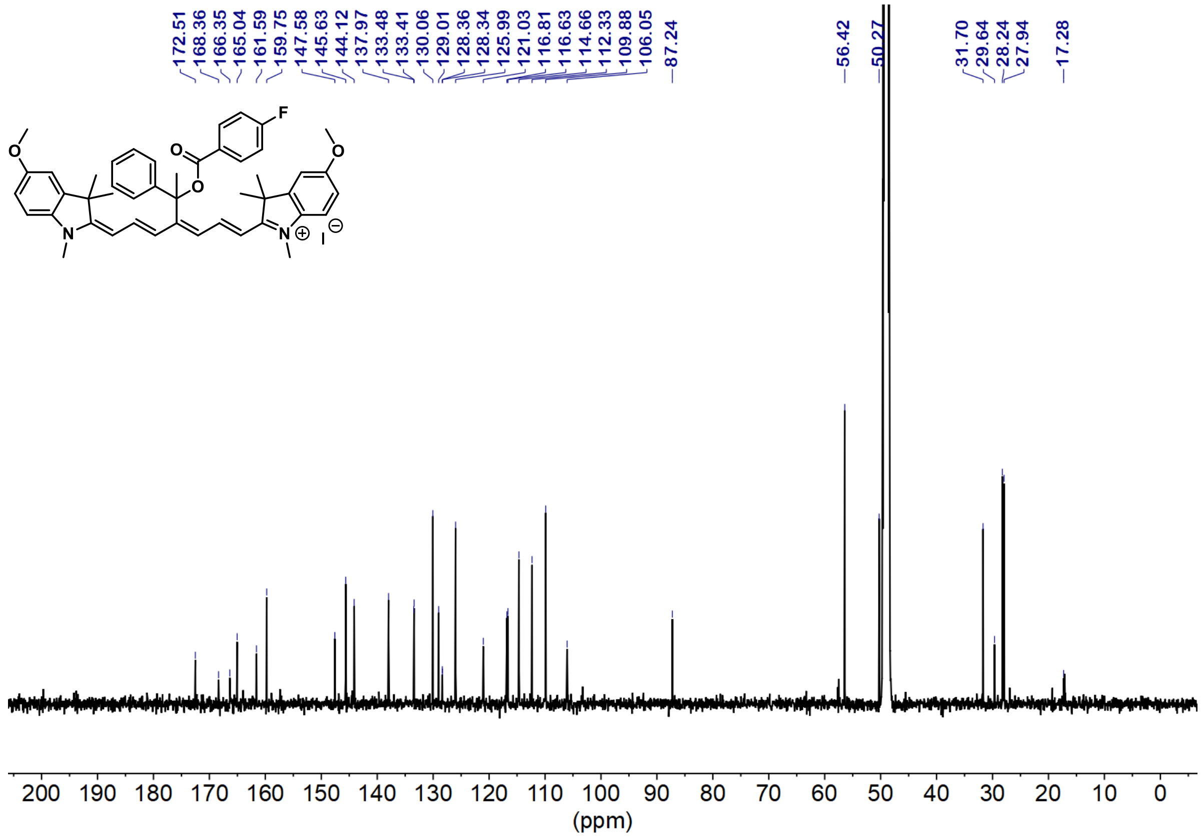

^{13}C NMR (126 MHz, d_4-CD_3OD) of **1-Ph**

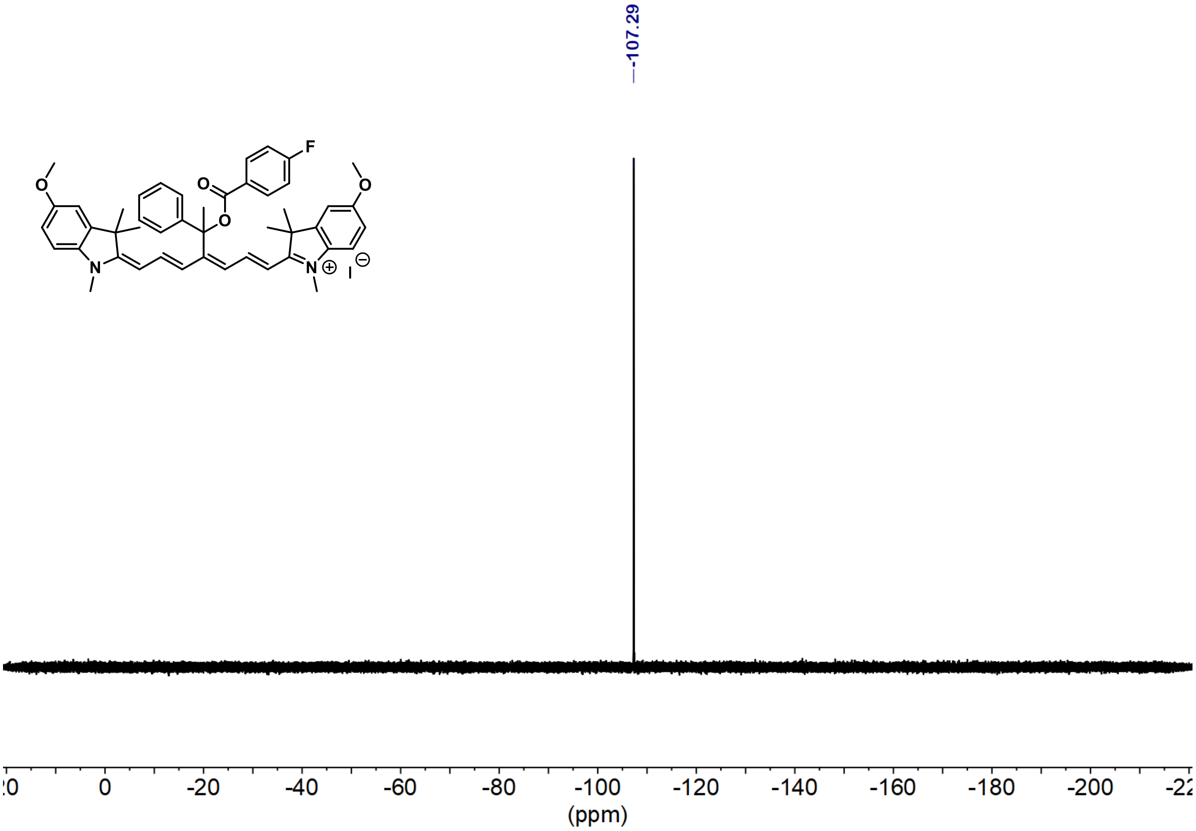

^{19}F NMR (471 MHz, d_4-CD_3OD) of **1-Ph**

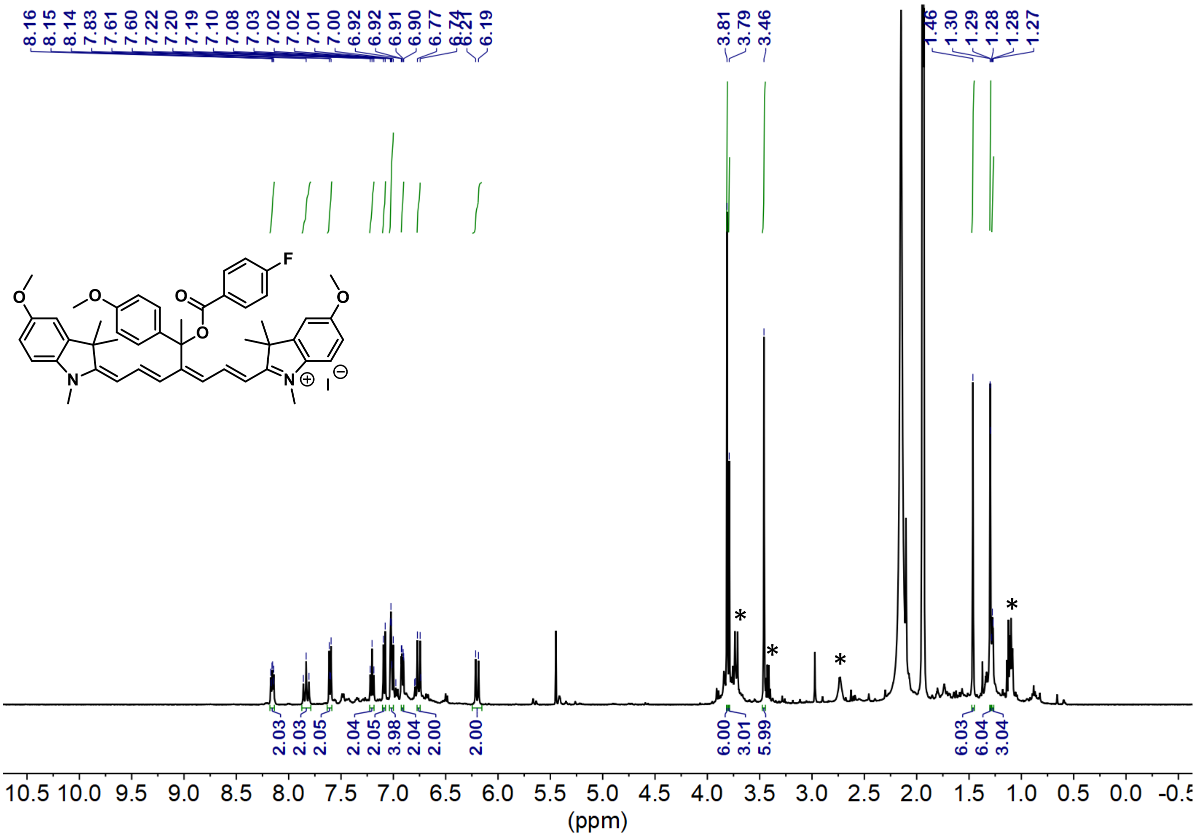

^{1}H NMR (500 MHz, d_3-CD_3CN) of **1-Ar**, *residual Et_3N/Et_3NH^+ from the deactivation of silica gel.

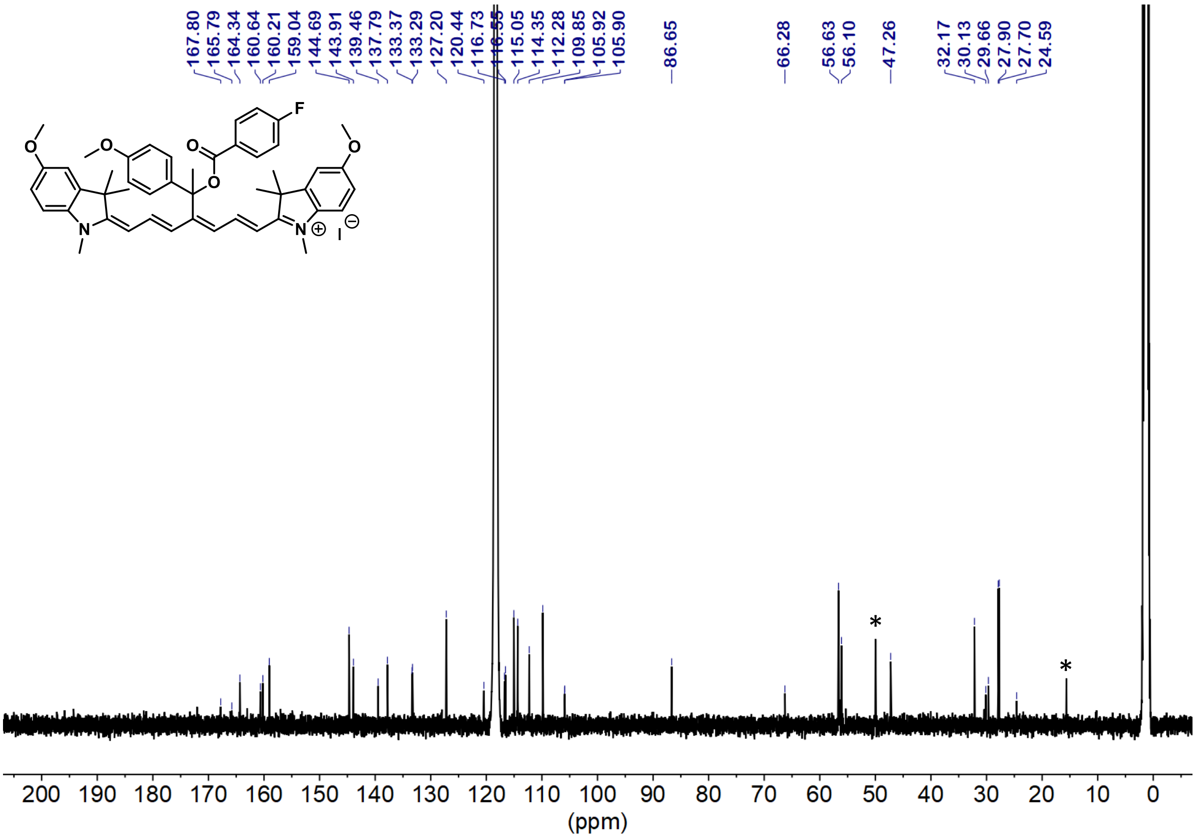

^{13}C NMR (126 MHz, d_3-CD_3CN) of **1-Ar**, *Et_3N/Et_3NH^+ residual Et_3N/Et_3NH^+ from the deactivation of silica gel.

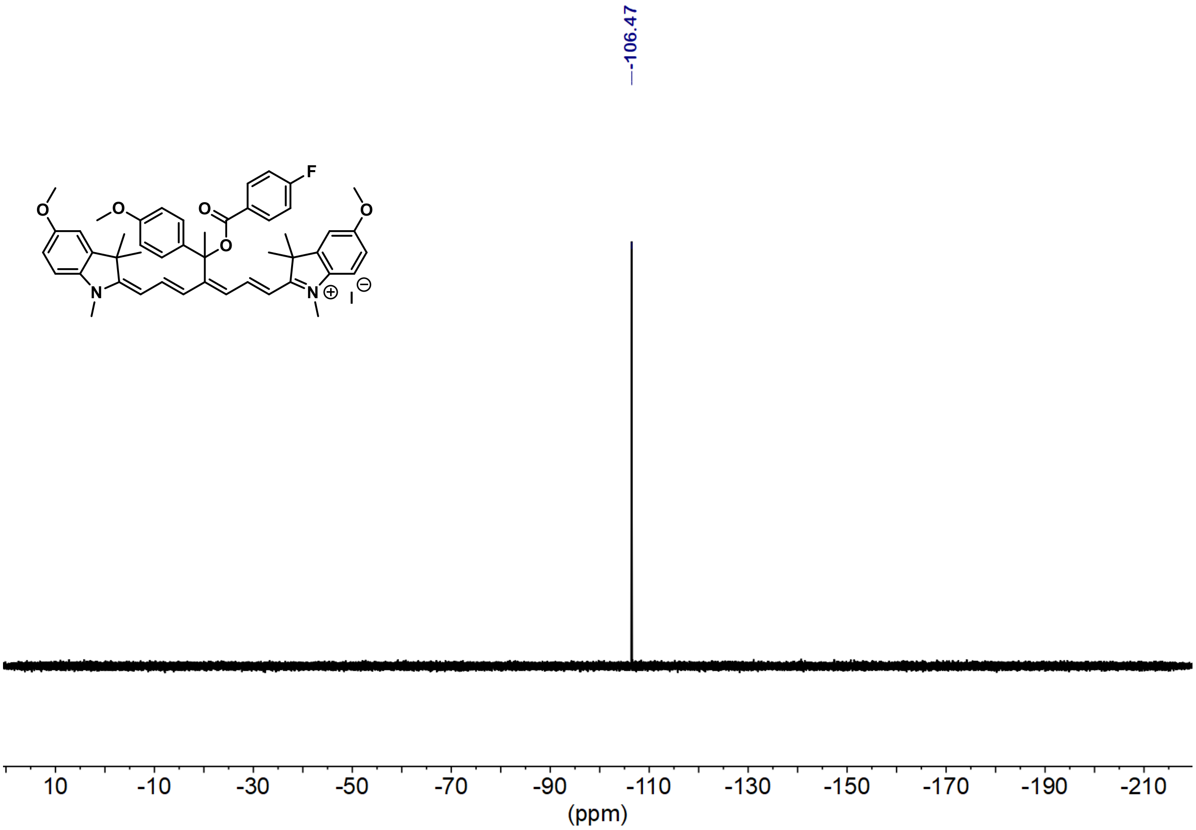

^{19}F NMR (471 MHz, d_3-CD_3CN) of **1-Ar**

UV-Vis Absorption and Emission Spectroscopy

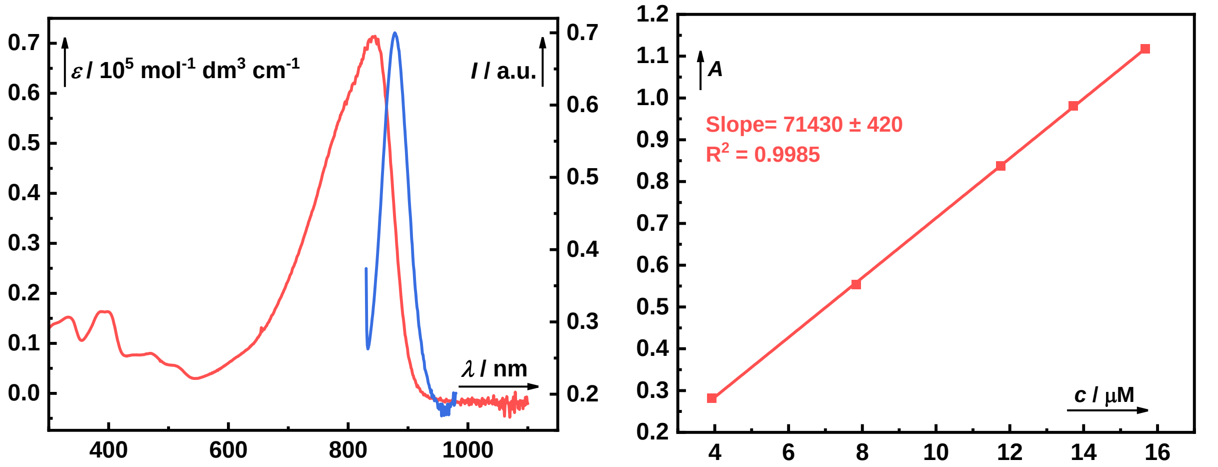

Figure S1. (left) UV-Vis absorption (red) and emission (blue) spectra of **1-Ph** in MeOH. (right) Dependence of absorption at λ_{max} on the concentration of **1-Ph** in MeOH (red).

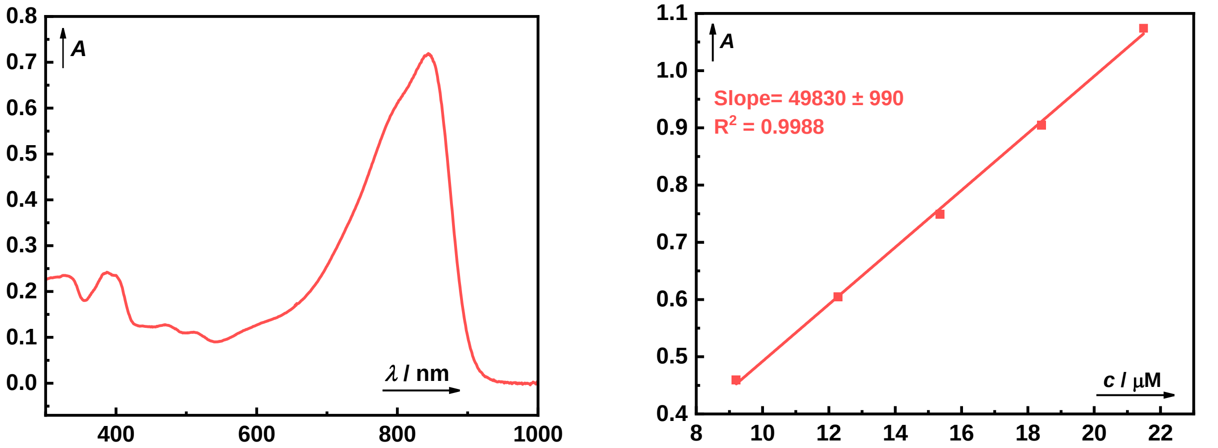

Figure S2. (left) UV-Vis absorption spectrum of **1-Ar** in MeOH. (right) Dependence of absorption at λ_{max} on the concentration of **1-Ar** in MeOH (red).

Plots of Photophysical and Photochemical Measurements

Irradiation and dark stability of the photocages followed by UV-vis Spectroscopy.

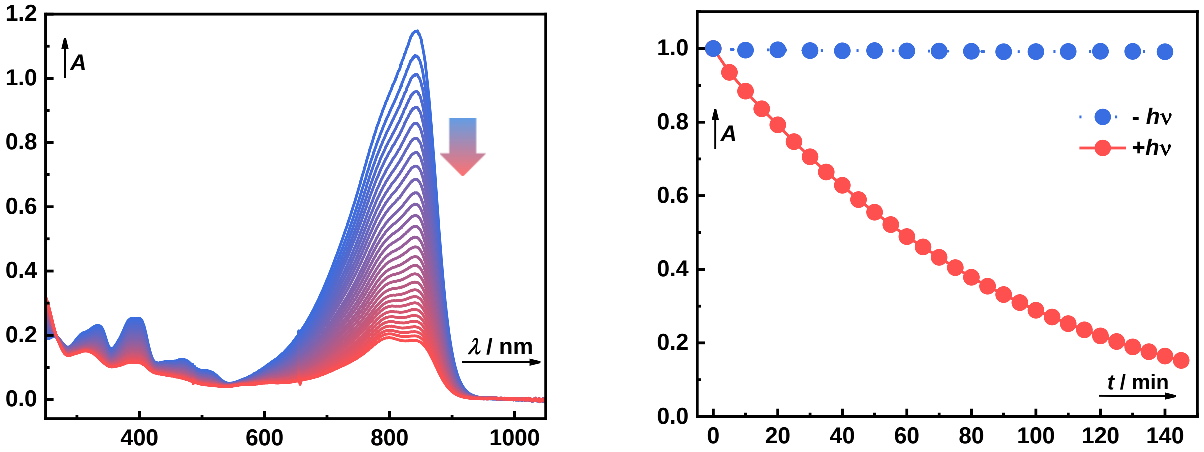

Figure S3. (left) Irradiation of **1-Ph** (A~1) at 820 nm in MeOH followed by UV-vis spectroscopy in 5-min intervals (blue to red). (right) Kinetic traces measured at λ_{max} in dark (blue) and under irradiation at 820 nm (red) in MeOH.

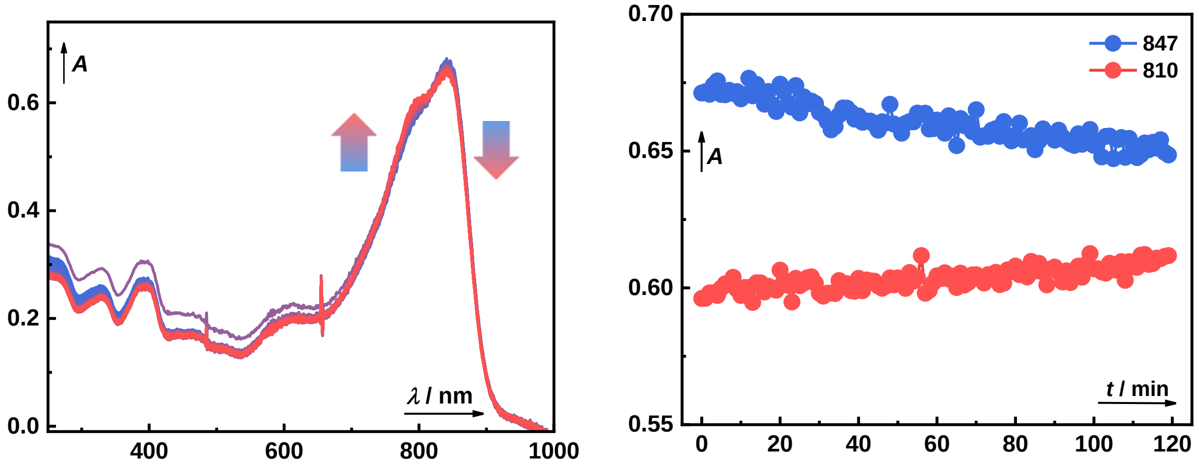

Figure S4. (left) Thermal decomposition of **1-Ar** (A~1) in MeOH in the dark followed by UV-vis spectroscopy in 10-min intervals (blue to red). (right) Kinetic traces measured at λ_{max}=847 nm (blue) and λ_{max}=810 nm (red).

Quantum yields of decomposition determined by UV-vis spectroscopy.

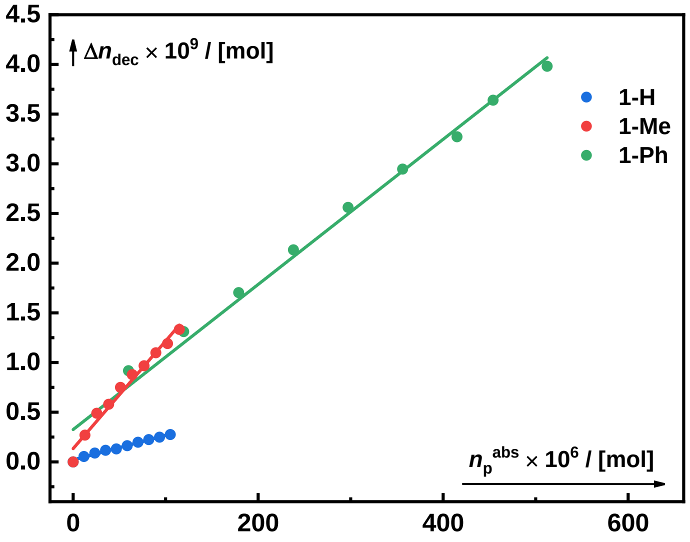

Figure S5. Quantum yield of decomposition of compounds **1-H, 1-Me** and **1-Ph** in MeOH.

Irradiation and dark stability of the photocages followed by NMR spectroscopy.

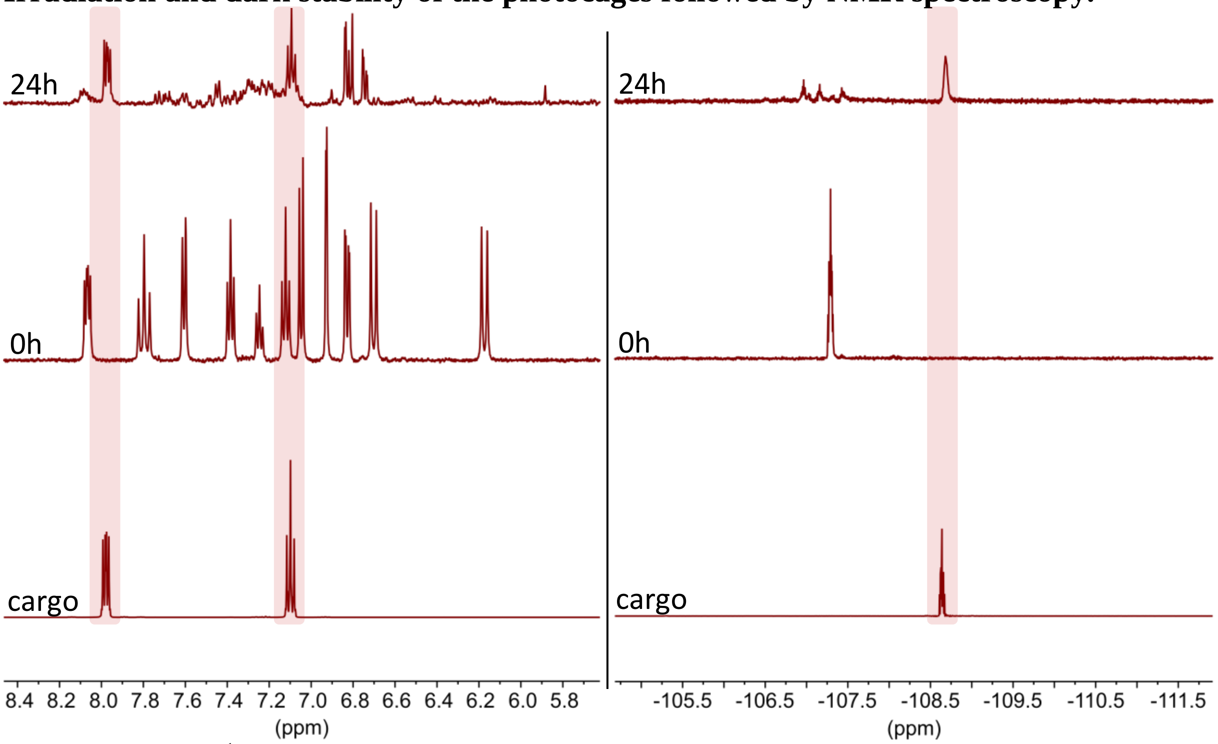

Figure S6. (left) ^{1}H NMR spectra of irradiation of **1-Ph** with 820 nm LEDs under ambient conditions, 4-fluorobenzoic acid release depicted in red. (right) ^{19}F NMR spectra of irradiation of **1-Ph** with 820 nm LEDs under ambient conditions, 4-fluorobenzoic acid release depicted in red.

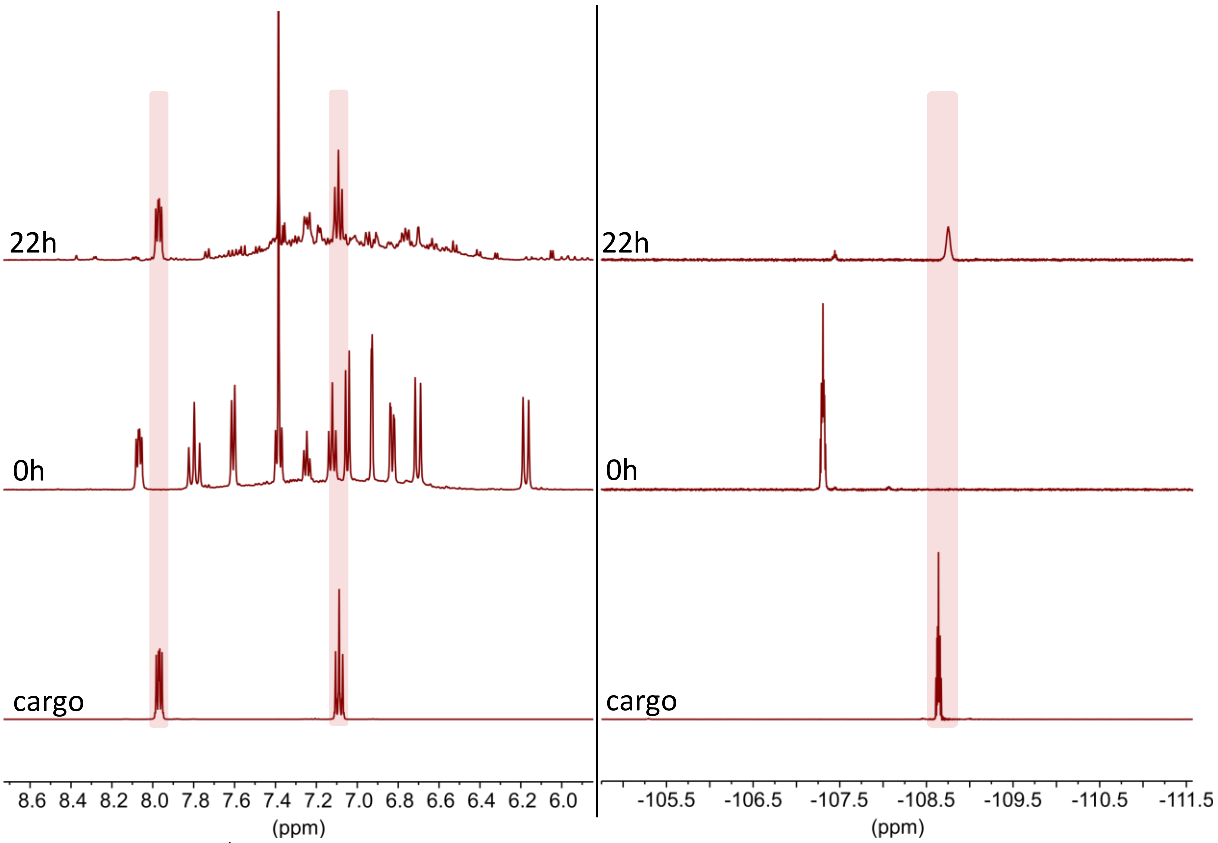

Figure S7. (left) ^{1}H NMR spectra of irradiation of **1-Ph** with 810 nm LEDs under O_2-free conditions, 4-fluorobenzoic acid release depicted in red, formation of new cyanine depicted in green. (right) ^{19}F NMR spectra of irradiation of **1-Ph** with 810 nm LEDs under O_2-free conditions, 4-fluorobenzoic acid release depicted in red.

Determination of Activation Parameters – Eyring Analysis by ^{1}H NMR Spectroscopy.

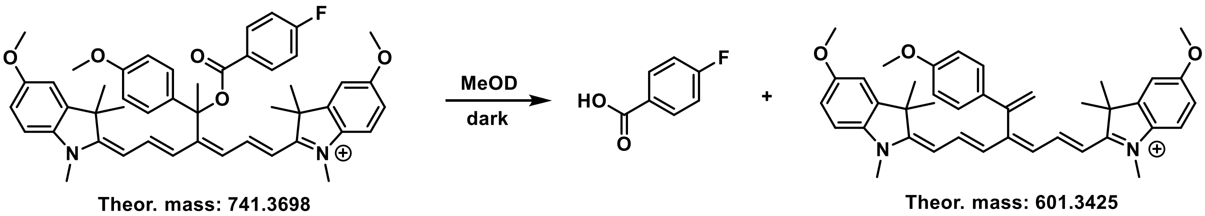

Figure S8. Scheme of thermal decomposition of **1-Ar** to eliminated product confirmed by HRMS and NMR spectroscopy.

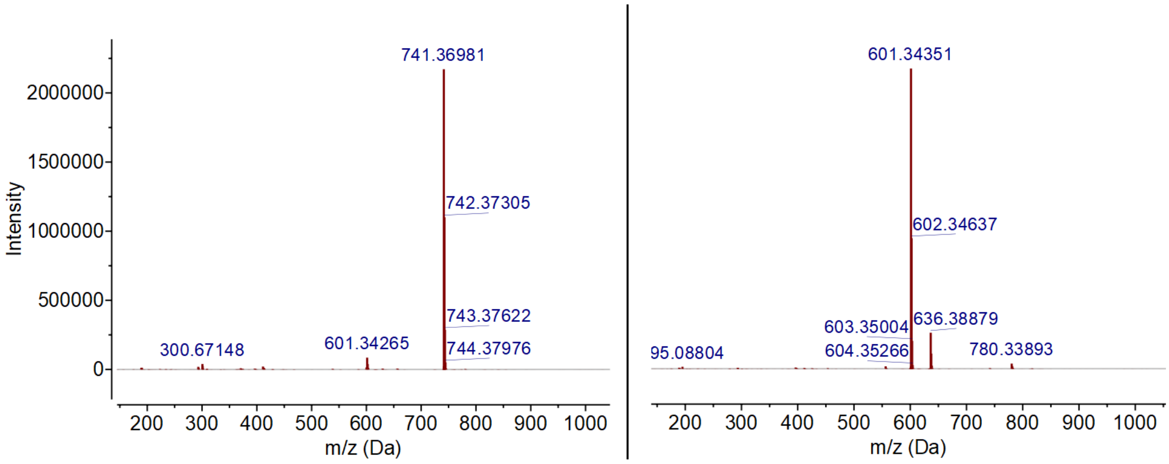

Figure S9. HRMS spectrum of **1-Ar** at t = 0 s (left), HRMS spectrum of **1-Ar** in MeOD, at t = 90 min. at 47.5°C (right).

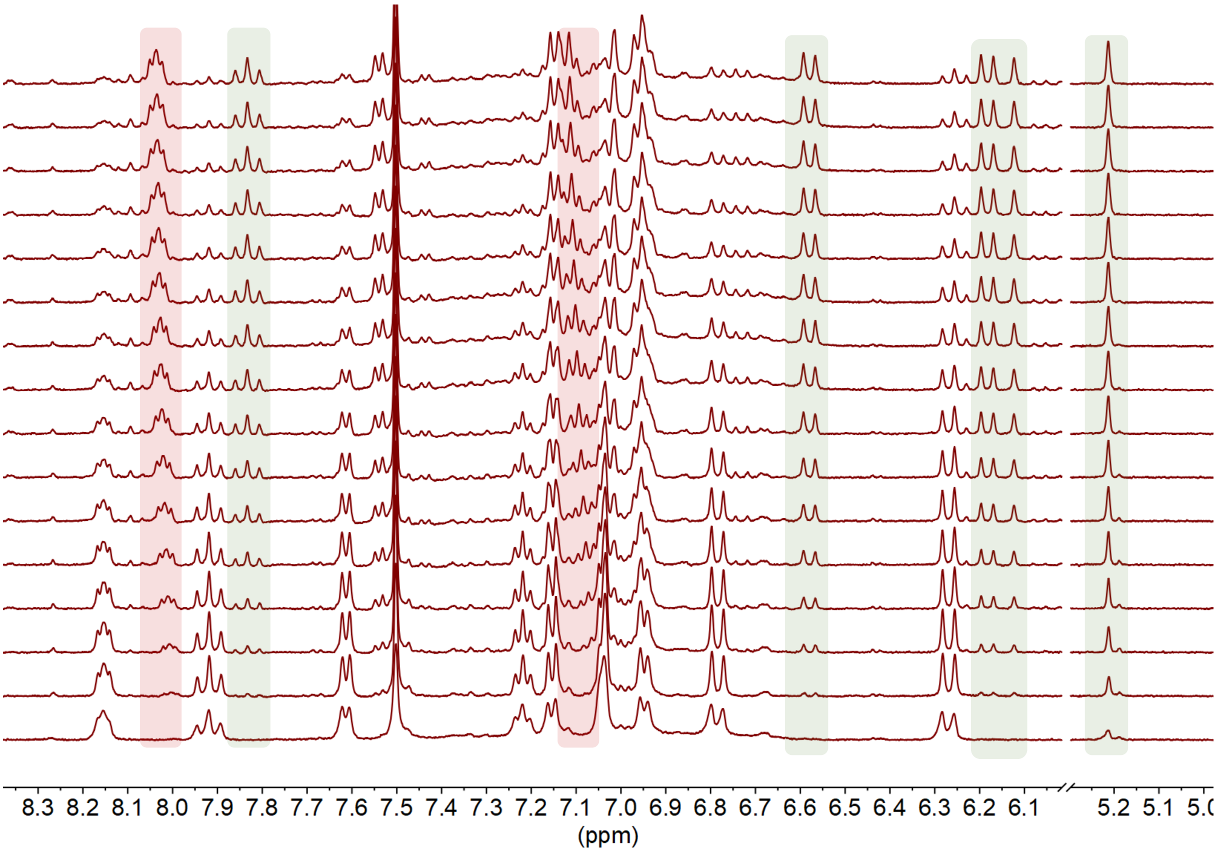

Figure S10. ^{1}H NMR spectra of **1-Ar** in MeOD in dark measured at 47.5°C measured in 366 s intervals. 4-Fluorobenzoic acid release depicted in red, formation of new cyanine depicted in green.

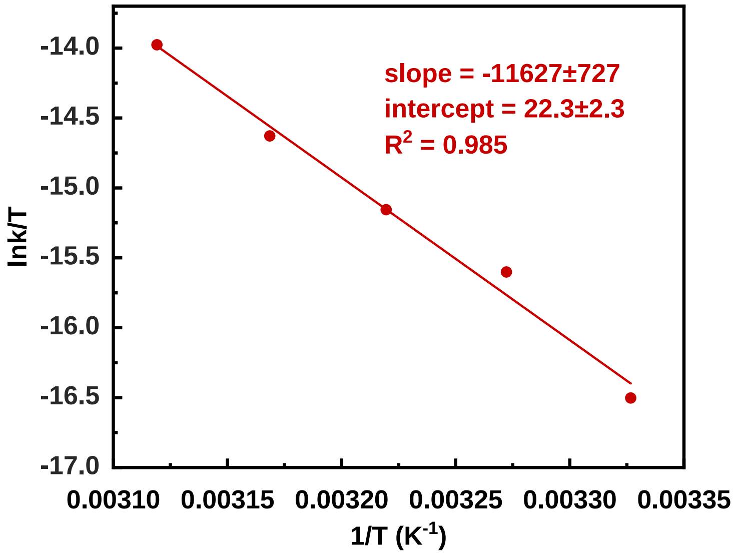

Figure S11. Eyring plot of solvolysis of **1-Ar** methanol-d_4.

Additional experiments

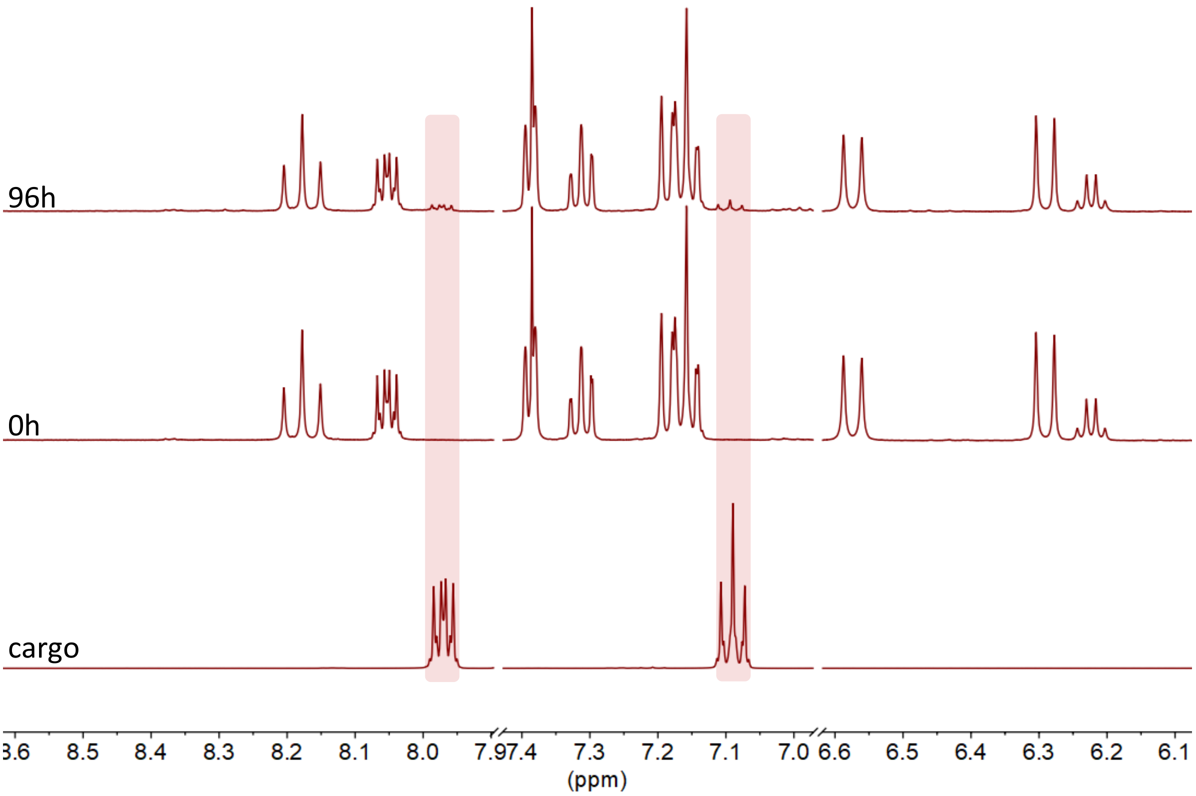

Figure S12. ^{1}H NMR spectra of irradiation of **1-H** with 810 nm LEDs under O_2-free conditions, 4-fluorobenzoic acid release depicted in red.

Irradiation Setups

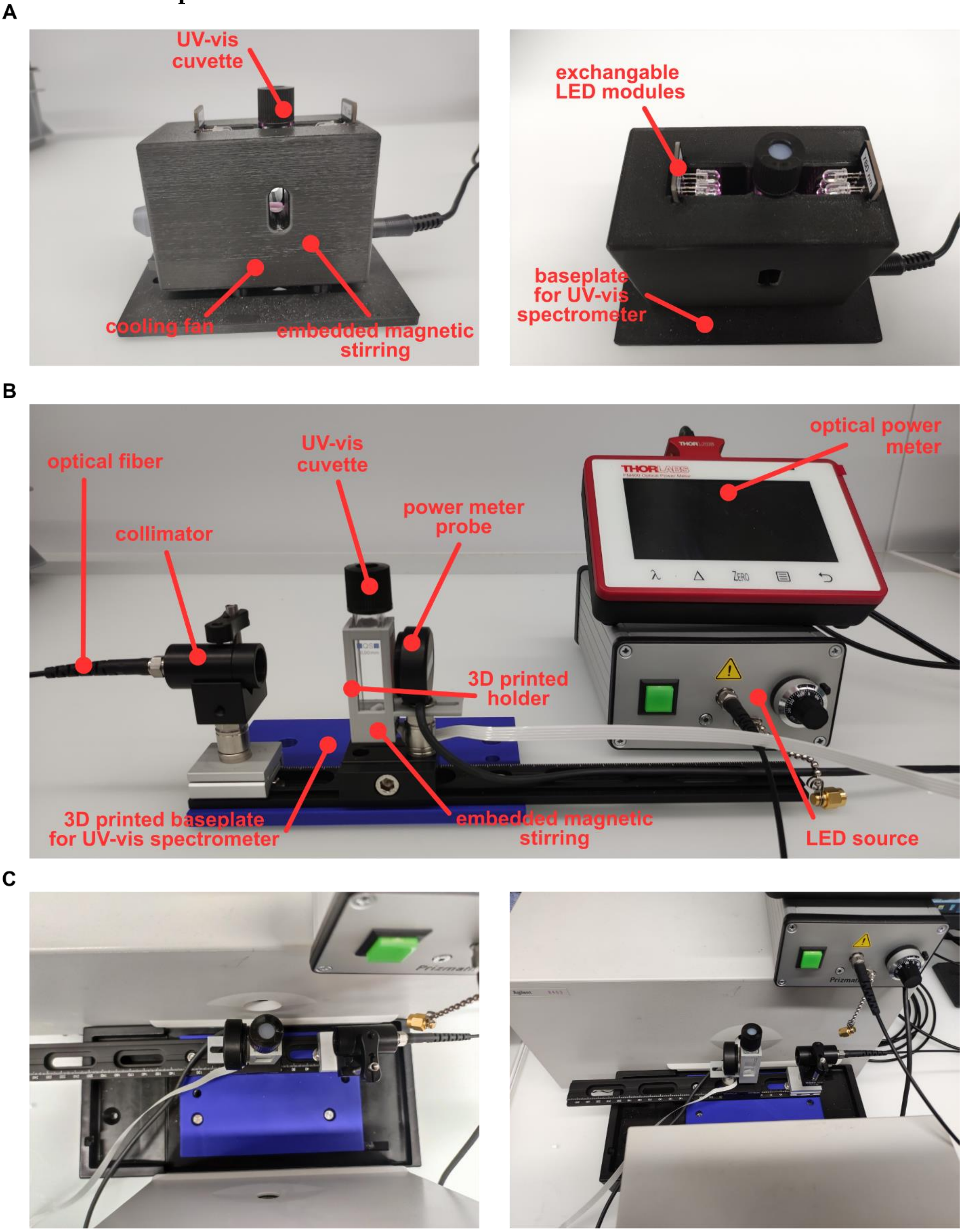

Figure S13. A) Irradiation module with magnetic stirring and exchangeable LED modules. B) In-house built irradiation setup with collimated light beam (780 nm) with magnetic stirring and coupled to optical power meter. C) Setup from (B) mounted inside a UV-vis spectrometer.

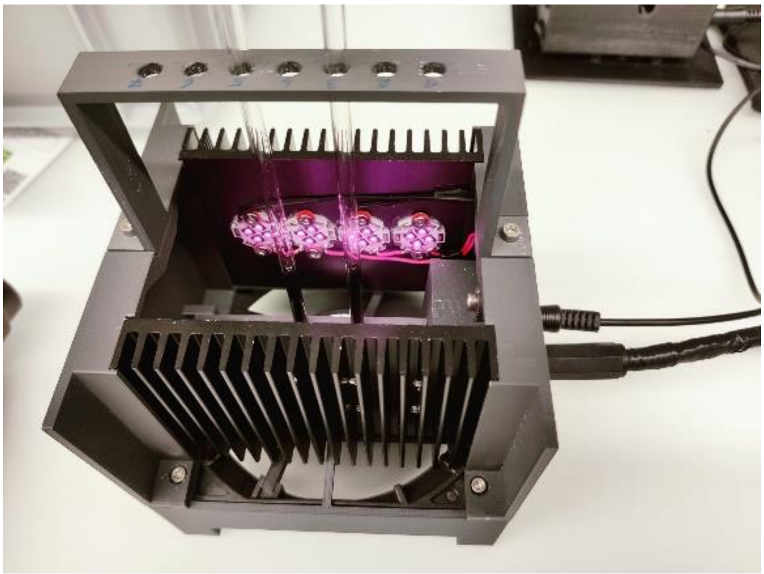

Figure S14. Device for irradiation of NMR tubes (810 nm LEDs, ~300 mW cm^{-2}, at a fixed distance of ~3 cm, cooled by a fan at 1200 rpm).

Calculations

General Remarks.

All calculations were performed with Gaussian16 rev. C.02 suite of electronic structure programs.[6] The ground state geometries of the potential energy minima were optimized at B3LYP/6-31G(d) level of theory. To reduce computational cost and avoid overestimation of energies resulting from steric repulsion, the structures of cyanine **1** were simplified by omitting the methoxy substituents on the heterocycles and by substituting the carboxylate payload by a hydrogen atom (Scheme S1).

The nature of all stationary points was verified by a frequency calculation. The wavefunction stability of cyanine carbenium ions **2** was tested. If an instability was found, the broken spin-symmetry Kohn-Sham wavefunctions were computed, in which the spatial symmetries of the α and β MOs are destroyed. Such wavefunction was then used to optimize the geometry of the molecule. We denote such calculations here as BS-DFT. The carbocation/carbenium ion stabilization energies (CSE) were calculated using isodesmic reactions with **1-Ref** and **2-Ref** (see Table S1) as a reference. Solvation energies were calculated as single-point energies on optimized carbenium ions **2** using BS-DFT with SMD solvation model (solvent = water).

The single point energies were then calculated with various DFT functionals and the cc-pVTZ basis set. The reported energies (at 0 K) given in kcal mol^{-1} represent the sum of the total electronic energy and the unscaled zero-point energy correction.

To explore the excited state potential energy surfaces, the payload in cyanines **1** was replaced by pyridinium as the payload (Scheme S1) to mitigate the Coulombic attractions during the heterolytic bond cleavage (see the main text for further discussion). The potential energy surface scans for the S_1 excited state were performed usin TD-CAM-B3LYP/6-31G(d) level of theory to avoid spurious intrusion of charge-transfer states as we observed with B3LYP functional. Scans in S_1 states were carried out along the C_{meso}–N_{pyr} bond stretching coordinate in **1**, while all other coordinates were relaxed in the optimization. The transition states were optimized at the same level of theory starting from the potential energy surface scan maxima and verified by a frequency calculation (one imaginary force constant). Representations of the structures in Figures S15-16 were produced using CYLview20.[7]

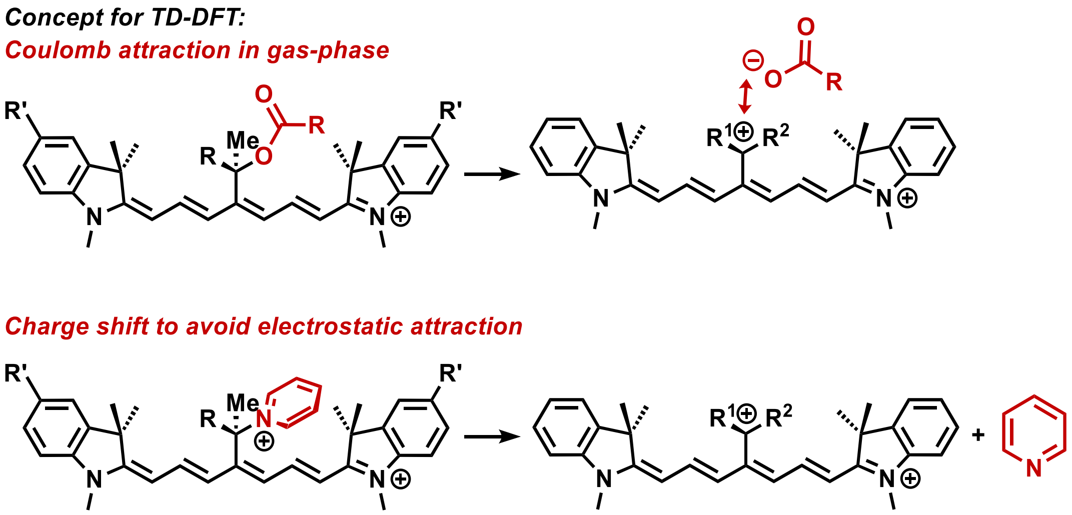

Scheme S1. Concept used for S_1 excited state TD-DFT calculations of **1**.

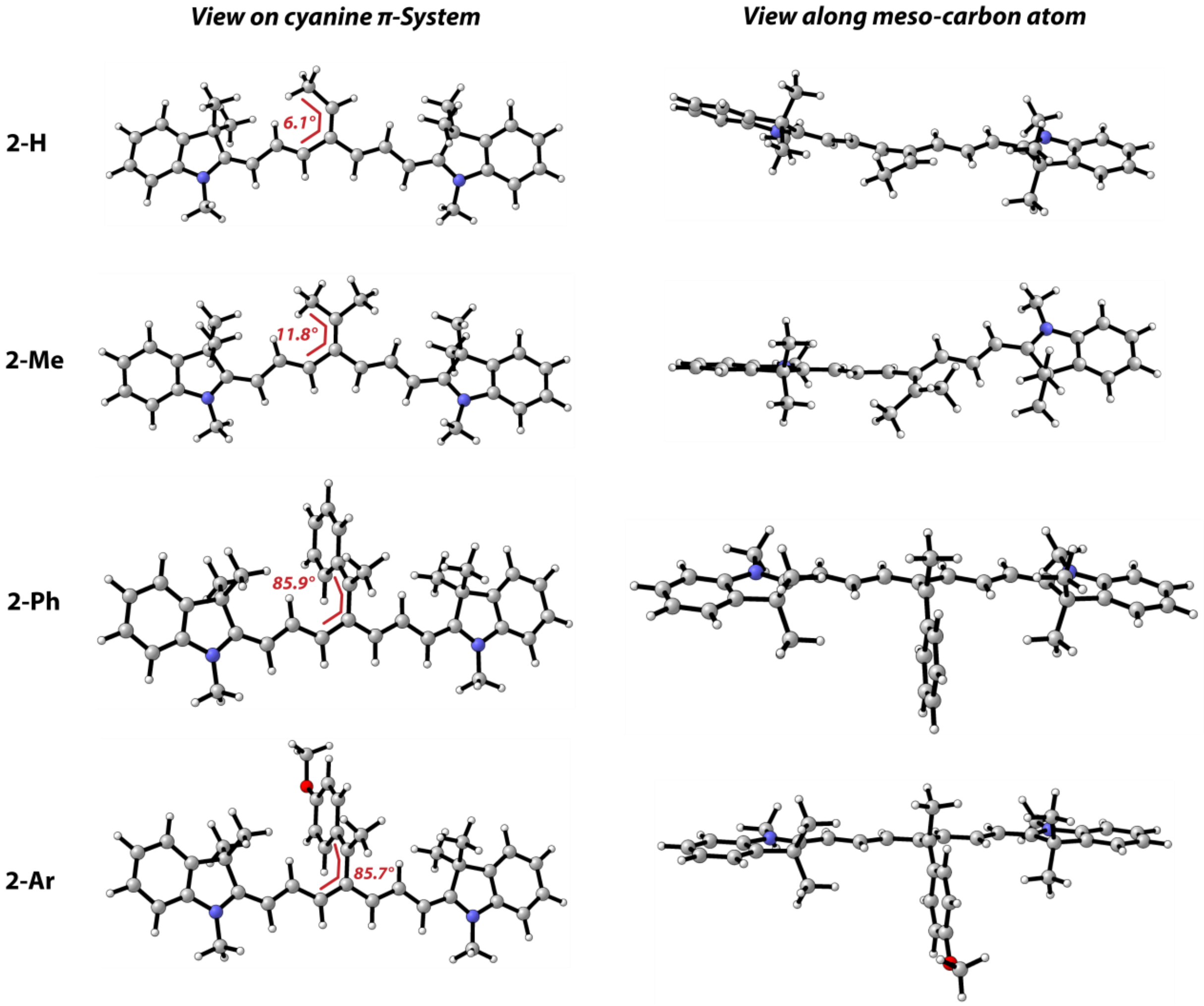

Figure S15. Optimized geometries of **2** at the BS-B3LYP/6-31G(d) level of theory. All geometries are shown in two perspectives to indicate the differences in the dihedral angle between the C_{meso}-substituents and the Cy7 π-system. The torsion angle is highlighted in red.

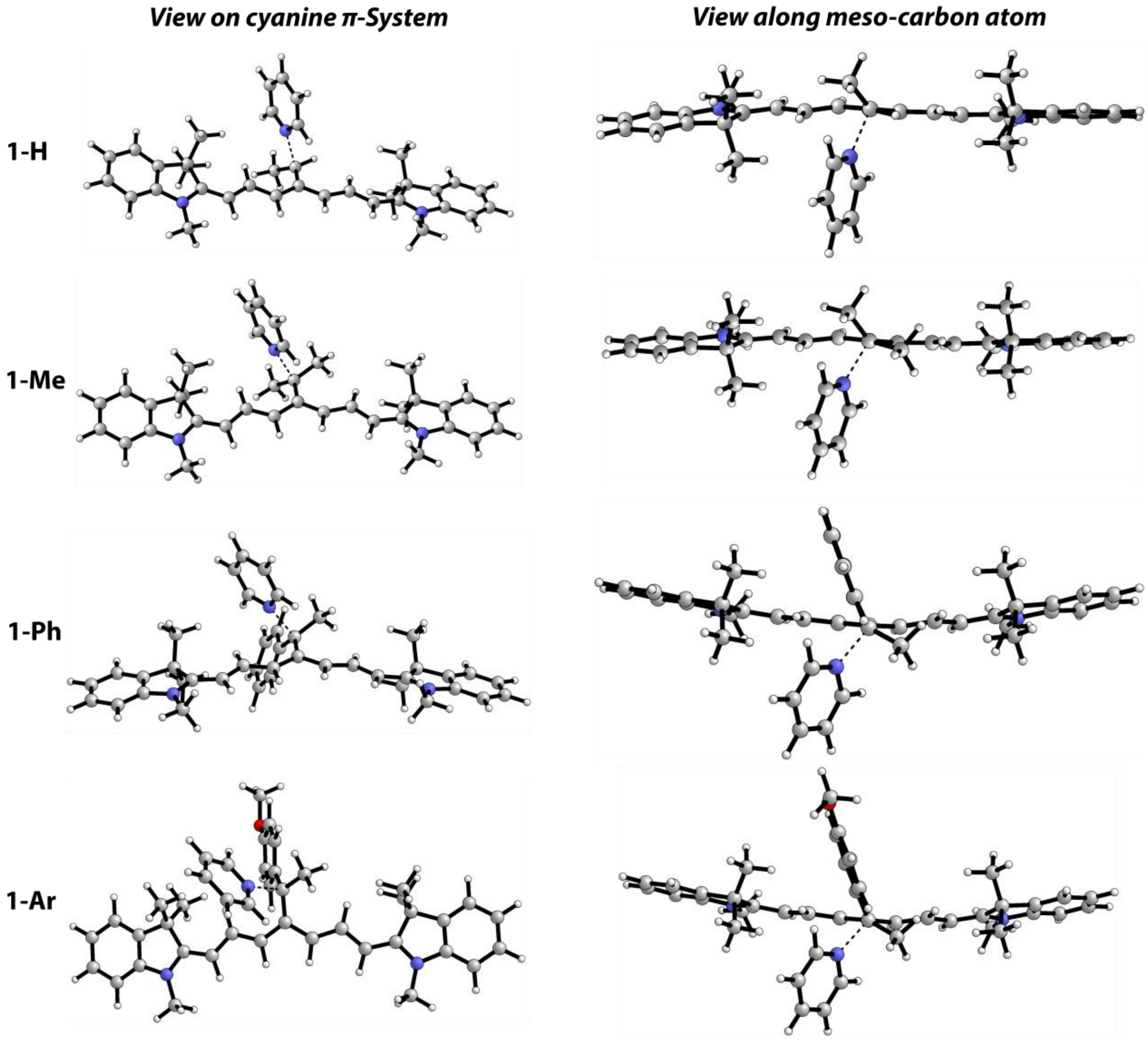

Figure S16. Optimized transition state geometries of **1** in the S_1 state at TD-CAM-B3LYP/6-31G(d) level of theory. All geometries are shown in two perspectives.

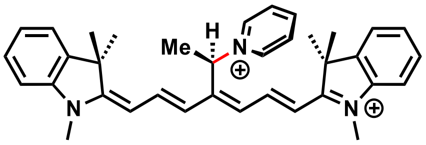

1-H

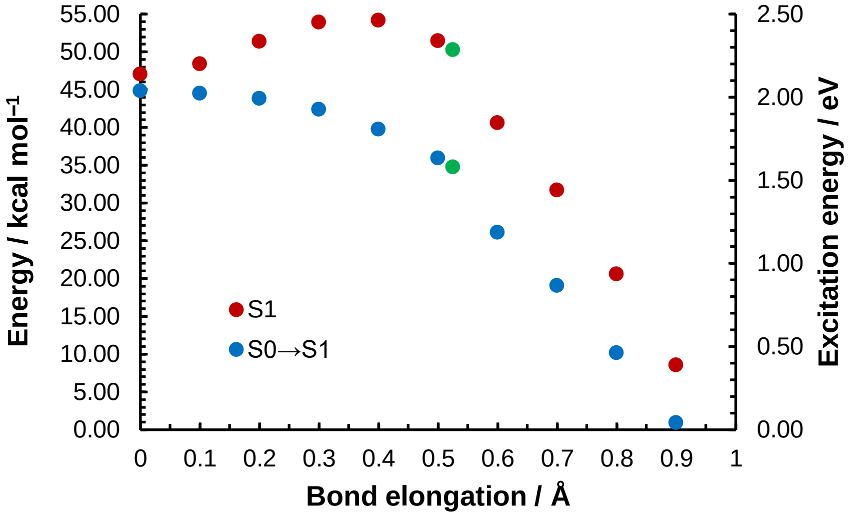

Figure S17. The S_1 excited state potential energy surface scans along the C_{meso}–N_{pyr} bond stretching (highlighted in the scheme) coordinate in **1-H** in (red) and the excitation energy S0→S1 in eV (blue). The optimized transition state is indicated in green. TD-CAM-B3LYP/6-31G(d) level of theory. The bond lengths in the S_1 state is $d_{C–N}$ = 1.52 Å.

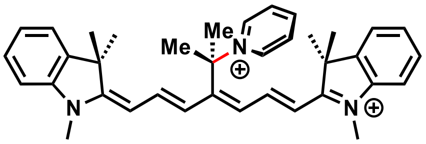

1-Me

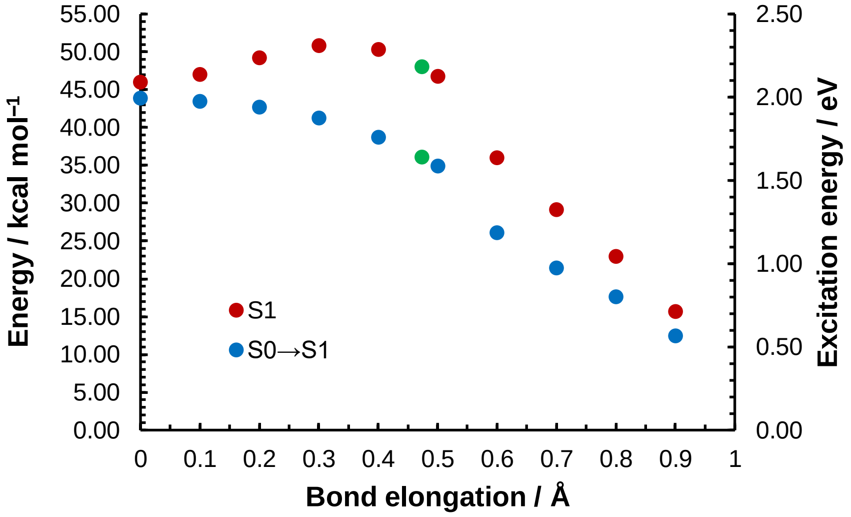

Figure S18. The S_1 excited state potential energy surface scans along the C_{meso}–N_{pyr} bond stretching (highlighted in the scheme) coordinate in **1-Me** in (red) and the excitation energy S0→S1 in eV (blue). The optimized transition state is indicated in green. TD-CAM-B3LYP/6-31G(d) level of theory. The bond lengths in the S_1 state is $d_{C–N}$ = 1.54 Å.

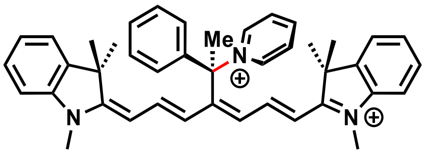

1-Ph

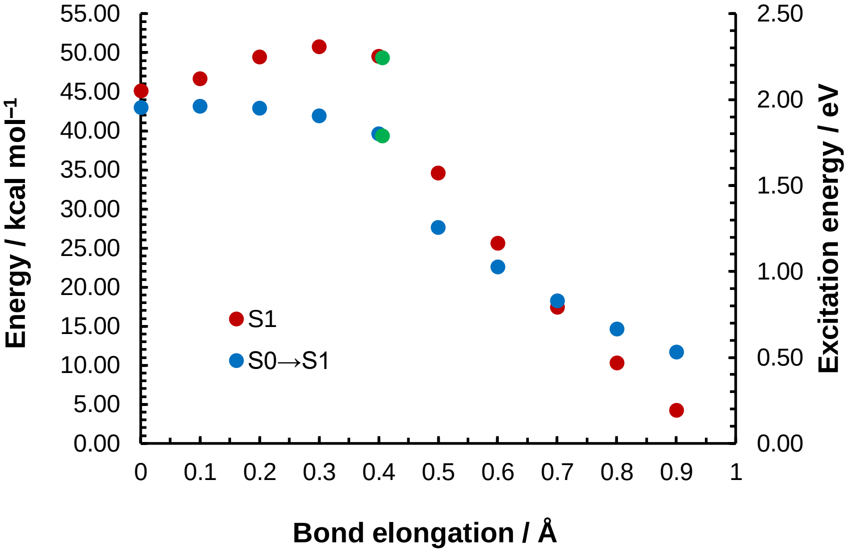

Figure S19. The S_1 excited state potential energy surface scans along the C–N bond stretching (highlighted in the scheme) coordinate in **1-Ph** in (red) and the excitation energy S0→S1 in eV (blue). The transition state is indicated in green. TD-CAM-B3LYP/6-31G(d) level of theory. The optimized bond lengths in the S_1 state is $d_{C–N}$ = 1.54 Å.

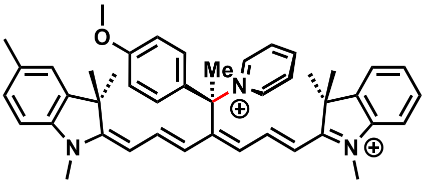

1-Ar

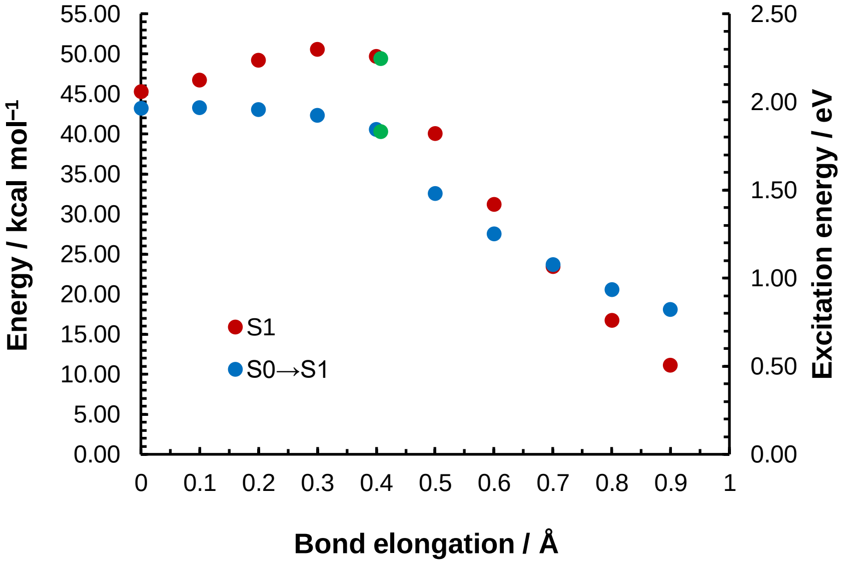

Figure S20. The S_1 excited state potential energy surface scans along the C–N bond stretching (highlighted in the scheme) coordinate in **1-Ar** in (red) and the excitation energy S0→S1 in eV

(blue). The transition state is indicated in green. TD-CAM-B3LYP/6-31G(d) level of theory. The optimized bond lengths in the S_1 state is $d_{C–N}$ = 1.55 Å.

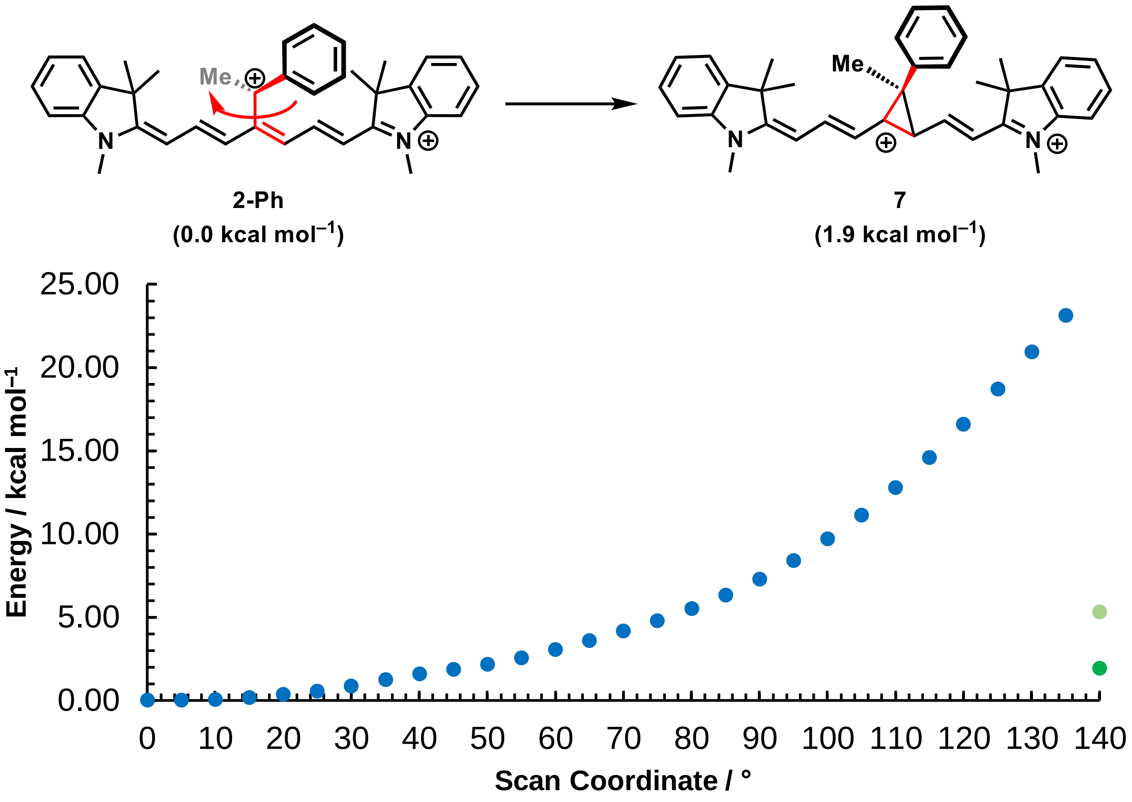

Figure S21. The ground state potential energy surface scan (blue and light green dots) along the dihedral angle coordinate (highlighted red) in **2-Ph**. The energy of the fully optimized closed ring structure **7** is shown as the dark green data point. B3LYP/6-31G(d) level of theory. The value of the torsion angle in optimized **2-Ph** (the 0° of the scanned coordinate) is 94.13°.

Table S1. Calculated cation/carbenium ion stabilization energies (CSE) of the hydride transfer between **1-Ref**/**2-Ref** and **1**/**2** in the ground state at different levels of theory using the isodesmic reaction shown below. Calculations were performed using the cc-pVTZ basis-set on (BS)-B3LYP/6-31G(d) optimized geometries. Energies are given at 0K including unscaled ZPVEs.

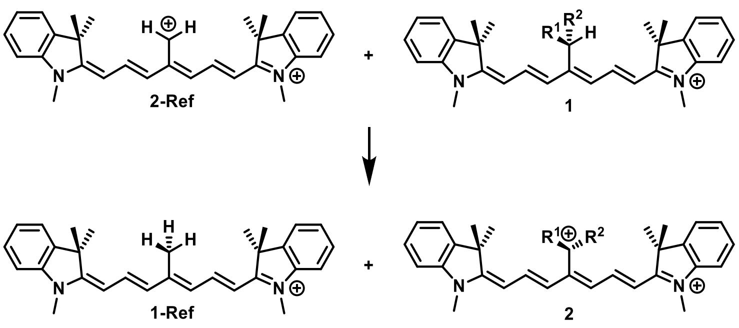

			CSE / kcal mol^{-1}		
**Compound**	**R^1**	**R^2**	**BMK**	**M06-2X**	**wB97XD**
**1-H**	H	Me	–3.96	–4.25	–3.92
**1-Me**	Me	Me	–9.07	–10.52	–9.34
**1-Ph**	Me	Ph	–10.66	–9.89	–8.53
**1-Ar**	Me	4-MeOPh	–22.18	–21.50	–17.64
**1e**	H	Ph	–4.43	–3.08	–2.05
**1f**	H	4-MeOPh	–16.65	–15.26	–12.44

Table S2. Calculated cation/carbenium ion stabilization energies (CSE) using broken symmetry singlet energies ($E_{BS\text{-}singlet}$) and spin-contamination corrected singlet energies (E_{corr}) of the hydride transfer between **1-Ref**/**2-Ref** and **1**/**2** at (BS)-B3LYP/6-31G(d) level of theory. Calculations were performed using (BS)-B3LYP/6-31G(d) optimized geometries. We corrected the spin contamination by the triplet state in the broken symmetry Kohn-Sham wavefunction using equation below to obtain the corrected singlet energies ($E_{singlet}$)[8]. $E_{<S2>=0}$ is the BS-DFT energy, $\langle S^2 \rangle$ gives the expectation value of the total-spin operator of the broken-symmetry Kohn-Sham wavefunction, and $E_{<S2>=1}$ is the triplet state energy computed on the BS-B3LYP geometry. CSE's were subsequently calculated using the isodesmic reaction shown in Table S1. Energies are given at 0K including unscaled ZPVEs.

$$E_{singlet} = \frac{2E_{<S2>=0} - < S^2 > E_{<S2>=1}}{2 - < S^2 >}$$

				CSE / kcal mol^{-1}	
**Compound**	**R^1**	**R^2**	**<S^2>**	$E_{BS\text{-}singlet}$	E_{corr}
**1a**	H	Me	0.751	–4.45	–4.77
**1b**	Me	Me	0.120	–9.84	–11.00
**1c**	Me	Ph	0.269	–10.71	–11.14
**1d**	Me	4-MeOPh	0.483	–24.71	–30.42
**1e**	H	Ph	0.703	–8.27	–7.20
**1f**	H	4-MeOPh	0.767	–20.30	–22.55

Table S3. SMD solvation energies of carbenium ions **2**. Energies were calculated on BS-B3LYP/6-31G(d) level of theory.

Compound	Solvation Energy / kcal mol^{-1}
**2-Ref**	–121.02
**2-H**	–119.88
**2-Me**	–118.84
**2-Ph**	–116.38
**2-Ar**	–116.02

Table S4. Mulliken charges for C_{meso} carbon atom in **1** in the S_1 excited state for the energy minima and transition states of the C_{meso}–N_{pyr} bond stretch shown in Figures S15–16. Calculations were performed using TD-CAM-B3LYP densities on TD-CAM-B3LYP/6-31G(d) optimized geometries.

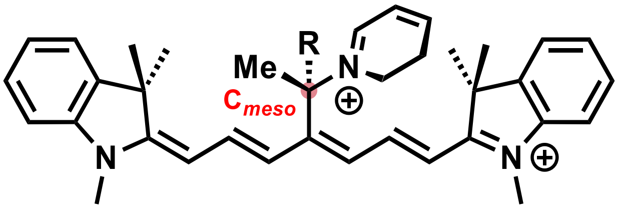

1

Compound	R^1	Transition state
**1-H**	–0.075	–0.107
**1-Me**	0.087	0.100
**1-Ph**	0.050	0.037
**1-Ar**	0.046	0.039

Table S5. Calculated cation/carbenium stabilization energies (CSE) of the hydride transfer between **1-Ref**/**2-Ref** and aryl-ethanes in the ground state at different levels of theory using the isodesmic reaction shown below. Calculations were performed using the cc-pVTZ basis-set on (BS)-B3LYP/6-31G(d) optimized geometries. Energies are given at 0K including unscaled ZPVEs.

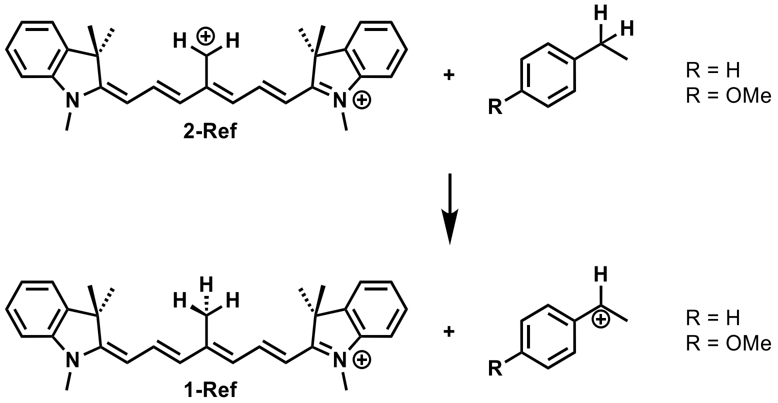

	CSE / kcal mol^{-1}		
**Compound**	**BMK**	**M06-2X**	**wB97XD**
**Ethylbenzene**	−42.68	−43.95	−40.50
**1-Ethyl-4-methoxybenzene**	−57.95	−58.53	−55.16

References

[1] Štacková, L.; Štacko, P.; Klán, P. *J. Am. Chem. Soc.* **2019**, *141*, 7155–7162.
[2] Mbera Ngale Efange, S.; Michelson, R. H.; Remmel, R. P.; Boudreau, R. J.; Dutta, A. K.; Freshler, A. *J. Med. Chem.* **1990**, *33*, 3133–3138.
[3] Janeková H., Russo M., Ziegler U., Štacko P. *Angew. Chem.Int. Ed.* **2022**, *61*, e202204391.
[4] Štacková, L.; Muchová, L.; Russo, M.; Slavíček, P.; Štacko, P.; Klán, P. *J. Org. Chem.* **2020**, *15*, 9776–9790.
[5] Rodgers, M. A. J. *J. Am. Chem. Soc.* **1983**, *105*, 6202–6205.
[6] Gaussian 16, Revision C.02, Frisch, M. J.; Trucks, G. W.; Schlegel, H. B.; Scuseria, G. E.; Robb, M. A.; Cheeseman, J. R.; Scalmani, G.; Barone, V.; Petersson, G. A.; Nakatsuji, H.; Li, X.; Caricato, M.; Marenich, A. V.; Bloino, J.; Janesko, B. G.; Gomperts, R.; Mennucci, B.; Hratchian, H. P.; Ortiz, J. V.; Izmaylov, A. F.; Sonnenberg, J. L.; Williams-Young, D.; Ding, F.; Lipparini, F.; Egidi, F.; Goings, J.; Peng, B.; Petrone, A.; Henderson, T.; Ranasinghe, D.; Zakrzewski, V. G.; Gao, J.; Rega, N.; Zheng, G.; Liang, W.; Hada, M.; Ehara, M.; Toyota, K.; Fukuda, R.; Hasegawa, J.; Ishida, M.; Nakajima, T.; Honda, Y.; Kitao, O.; Nakai, H.; Vreven, T.; Throssell, K.; Montgomery, J. A., Jr.; Peralta, J. E.; Ogliaro, F.; Bearpark, M. J.; Heyd, J. J.; Brothers, E. N.; Kudin, K. N.; Staroverov, V. N.; Keith, T. A.; Kobayashi, R.; Normand, J.; Raghavachari, K.; Rendell, A. P.; Burant, J. C.; Iyengar, S. S.; Tomasi, J.; Cossi, M.; Millam, J. M.; Klene, M.; Adamo, C.; Cammi, R.; Ochterski, J. W.; Martin, R. L.; Morokuma, K.; Farkas, O.; Foresman, J. B.; Fox, D. J. Gaussian, Inc., Wallingford CT, **2016**.

[7] CYLview20; Legault, C. Y. Université de Sherbrooke, **2020** (http://www.cylview.org)

[8] a) Yamaguchi, K.; Jensen, F.; Dorigo, A.; Houk, K. N. *Chem. Phys. Lett.* **1988**, *149*, 537–542.

b) Albright, T. R.; Arthur H. Winter, A. H. *J. Am. Chem. Soc.* **2015**, *137*, 3402–3410.

c) Isobe, H.; ,Takano, Y.; ,Kitagawa, Y.; ,Kawakami, T.; ,Yamanaka, S.; ,Yamaguchi, K.; Houk, K. N. *Mol. Phys.* **2002**, *100*, 717–727.
